# Recruitment of Armitage and Yb to a transcript triggers its phased processing into primary piRNAs in *Drosophila* ovaries

**DOI:** 10.1371/journal.pgen.1006956

**Published:** 2017-08-21

**Authors:** Radha Raman Pandey, David Homolka, Kuan-Ming Chen, Ravi Sachidanandam, Marie-Odile Fauvarque, Ramesh S. Pillai

**Affiliations:** 1 Department of Molecular Biology, University of Geneva, Geneva, Switzerland; 2 Department of Oncological Sciences, Icahn School of Medicine at Sinai, New York, New York, United States of America; 3 Biosciences and Biotechnology Institute of Grenoble (BIG), CEA-DRF-BIG-BGE, INSERM U1038, Univ. Grenoble Alpes, Grenoble, France; University of Cambridge, UNITED KINGDOM

## Abstract

Small RNAs called PIWI -interacting RNAs (piRNAs) are essential for transposon control and fertility in animals. Primary processing is the small RNA biogenesis pathway that uses long single-stranded RNA precursors to generate millions of individual piRNAs, but the molecular mechanisms that identify a transcript as a precursor are poorly understood. Here we demonstrate that artificial tethering of the piRNA biogenesis factor, Armi, to a transcript is sufficient to direct it into primary processing in *Drosophila* ovaries and in an ovarian cell culture model. In the fly ovarian somatic follicle cells, the transcript becomes cleaved in a stepwise manner, with a 5′→3′ directionality, liberating U1-containing ~24 nt piRNAs that are loaded into Piwi. Although uridines are preferred for generation of piRNA 5′ ends, processing takes place even in their absence, albeit at a lower efficiency. We show that recombinant Armi has 5′→3′ helicase activity, and mutations that abolish this activity also reduce piRNA processing in vivo. Another somatic piRNA pathway factor Yb, an interactor of Armi, is also able to trigger piRNA biogenesis when tethered to a transcript. Tethering-mediated primary piRNA biogenesis is also functional in the fly ovarian germline and loads all the three PIWI proteins present in this environment. Our study finds a broad correlation between piRNA processing and localization of the tethered factors to the cytoplasmic perinuclear ribonucleoprotein granules called germline nuage or somatic Yb bodies. We conclude that transcripts bound by Armi and Yb are identified as piRNA precursors, resulting in localization to cytoplasmic processing granules and their subsequent engagement by the resident piRNA biogenesis machinery.

## Author summary

PIWI-interacting RNAs (piRNAs) are 24–30 nucleotide (nt) small RNAs that are exclusively expressed in animal germlines and are essential for suppression of transposable elements or ‘jumping genes’. Millions of piRNAs are produced from single-stranded transcripts that arise from large RNA polymerase II transcription units called piRNA clusters. Since they resemble other RNA pol II products like protein-coding mRNAs or non-coding RNAs, how piRNA precursors are selectively recruited to the biogenesis machinery is a big mystery. Here we demonstrate that artificial tethering of specific piRNA biogenesis factors to a reporter transcript is sufficient to identify it as a piRNA precursor in *Drosophila* ovaries and in an ovarian cell culture model. This results in fragmentation of the transcript into thousands of piRNAs, which are generated as a series of non-overlapping (phased) fragments that are loaded into PIWI proteins. Our study indicates that localization of the tethered factors to cytoplasmic perinuclear granules called nuage/Yb bodies is necessary for processing. Any mutations in the tethered factors that disrupt this sub-cellular localization, reduce processing. We believe that our strategy will allow generation of designer small RNAs to target almost any germline gene, and also provides a valuable tool to dissect the molecular mechanism of piRNA biogenesis.

## Introduction

Bulk of the eukaryotic genomes are composed of genetic material derived from mobile genetic elements called transposons. Their mobility within the genome can cause mutations or deletions, impacting genome integrity [1]. Given the diversity of transposable elements within any genome, small RNAs are used to sequence-specifically identify and repress transposons in organisms ranging from plants to animals. In animals, this task is entrusted with a set of gonad-specific 24–30 nucleotide (nt) small RNAs called PIWI-interacting RNAs (piRNAs) that associate with the PIWI clade Argonaute proteins [2]. The basic functional unit of the pathway consists of a small RNA bound to a PIWI protein, with the piRNA acting as a guide for the protein [3]. Some PIWI proteins function as small RNA-guided endonucleases (slicers), while others recruit histone or DNA methylation machineries to mediate transcriptional repression of target genomic loci [2]. Any impairment of the piRNA pathway results in de-repression of transposons and failure of germ cell development, causing infertility.

Biogenesis of piRNAs is a cytoplasmic event. Single-stranded transcripts that arise from large (50–100 kilobases) genomic regions called piRNA clusters [4] or transposon transcripts and some genic mRNAs are substrates for piRNA production. They are transcribed by RNA polymerase II [5] and exported [6] to the cytoplasm where piRNA biogenesis factors are enriched in cytoplasmic perinuclear granules called nuage [7, 8]. Primary processing is the default pathway that converts the piRNA precursors into thousands of piRNAs having a preference for a uridine at the 5′ end (U1-bias). Since piRNA precursors resemble other cellular transcripts in having features like a 5′ cap and a 3′ poly A tail [5], how they are specifically targeted to the primary processing pathway is largely unknown.

Studies conducted in the *Drosophila* ovarian system indicate a role for sequences within the precursor transcripts in the recruitment process. In the fly ovarian germline, presence of complementary binding sites for piRNAs in transcripts, and the resultant slicing by PIWI proteins Aubergine and Ago3 identifies it as a precursor. This enables the entry of one of the cleavage fragments into piRNA biogenesis that generates a series of phased/non-overlapping piRNAs [9–11]. In contrast, Piwi slicing is demonstrated to be not essential for piRNA biogenesis in the fly ovarian soma [12], and primary processing has to create piRNAs *de novo* from the precursors. Studies reveal that sequences termed piRNA-trigger sequences (PTSs) present at the 5′ end of piRNA-producing regions are necessary and sufficient for recruiting a transcript into the somatic piRNA biogenesis machinery [9, 13]. Deletion of such sequences from an endogenous precursor transcript impacts piRNA biogenesis from the locus [13]. Precise nature of these poorly conserved sequences, and how they work are currently not understood, but they are assumed to provide binding sites for piRNA biogenesis factor(s) to initiate primary piRNA processing.

In this study, we recruited piRNA biogenesis factors to a reporter transcript by artificial tethering and demonstrate its entry into primary processing using transgenic fly lines and an ovarian somatic cell culture model. Tethering of the conserved piRNA biogenesis factor Armitage (Armi) [14, 15] or the somatic piRNA biogenesis factor Yb [16, 17] to a transcript results in its identification as a piRNA precursor in the fly ovarian soma and a somatic cell culture model. This results in non-overlapping/phased conversion of the transcript into ~24 nt U1-containing primary piRNAs. A similar effect is seen when Armi is tethered to a transcript in the fly ovarian germline, with generated piRNAs entering all the three PIWI proteins present in this environment. We find that this ability to induce piRNA generation is broadly correlated with localization of the factors to cytoplasmic processing granules called nuage in germ cells [7] or Yb bodies in the soma [17, 18]. Our study reveals a strategy for generating artificial piRNAs capable of targeting any germline gene, and provides a useful tool for dissecting the molecular mechanisms of primary piRNA biogenesis.

## Results

### Armi tethering directs a transcript into primary piRNA biogenesis in the fly ovarian soma

**Fig 1:**
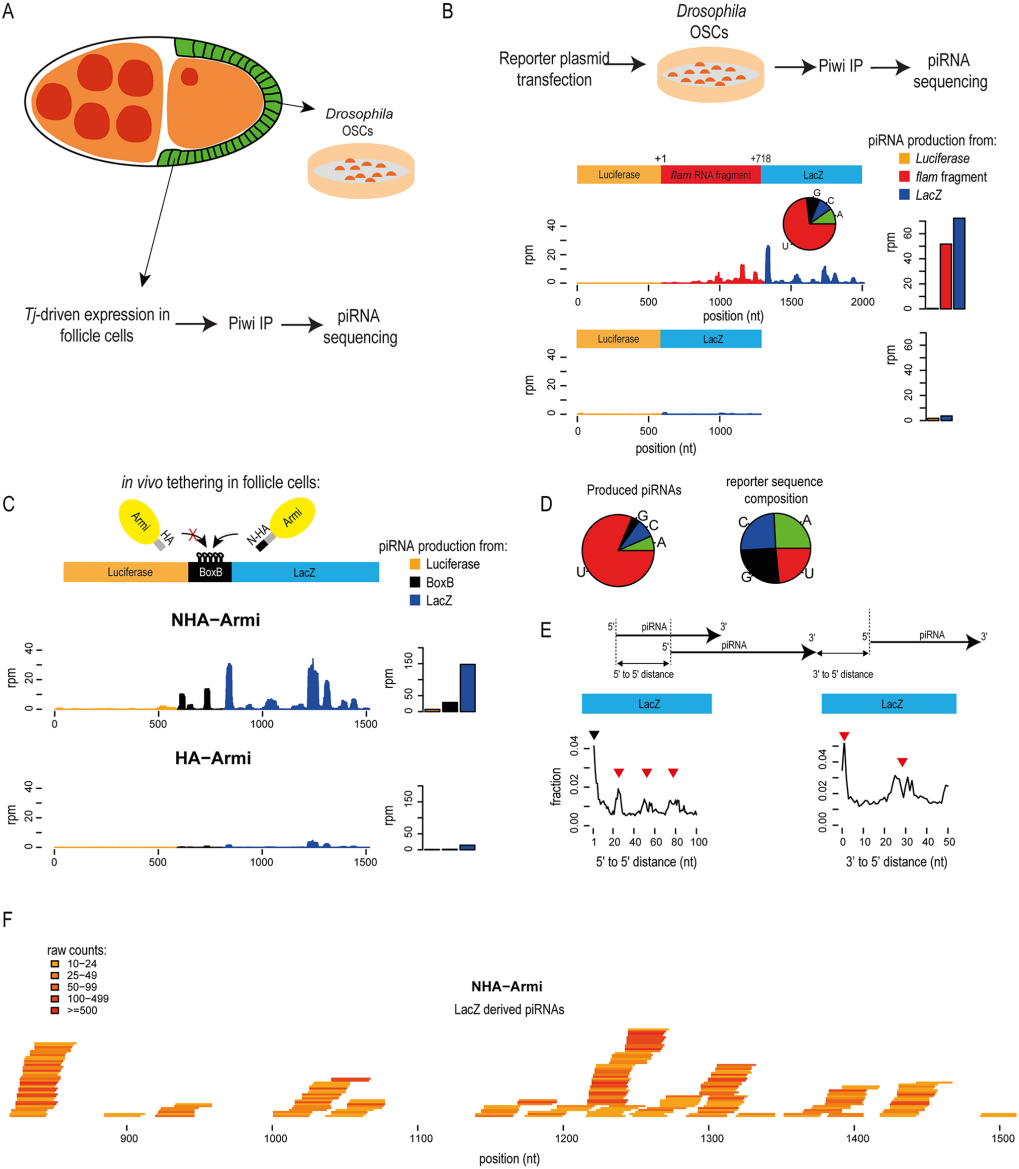
Artificial tethering of Armi to a transcript identifies it as a primary piRNA precursor in the *Drosophila* ovarian follicle cells. (A) Cartoon representing a *Drosophila melanogaster* egg chamber shows the single layer of somatic follicle cells enclosing the germline. Ovarian soma-specific co-expression of fusion proteins and the reporter RNA is achieved by the use of the *traffic jam* (*tj*)-GAL4 driver in the transgenic flies. Ovarian somatic cells (OSCs) is a cell culture model derived from the ovarian follicle cells.(B) A *cis*-acting element called piRNA-trigger sequence (PTS) from the *flamenco* (*flam*) cluster transcript drives conversion of the downstream reporter transcript into piRNAs that are loaded into Piwi in OSCs. The fragment used corresponds to 1–718 nt of the original cluster transcript. Read coverage (rpm) along the reporter and the total amount of piRNAs produced from individual transcript parts are shown. The 5′ nucleotide composition of reporter-derived piRNAs is shown as a pie chart. (C) BoxB reporter transcript was specifically co-expressed with either NHA-Armi or HA-Armi in the follicle cells of *Drosophila* ovary. Mapping of the generated piRNAs to the reporter is shown. (D) The 5′ end nucleotide preference of reporter-derived piRNAs indicates a strong U1-bias. Nucleotide composition of the reporter sequence is shown. (E) The 5′-to-5′ end-distances between the piRNAs produced upon NHA-Armi tethering is shown for the LacZ part of the transcript. Many piRNAs start at neighbouring nucleotides as demonstrated by high proportion of piRNAs with a 5′-to-5′ distance equal to one (black triangle). However, LacZ-derived piRNAs also show enriched distances which are multiple of the average piRNA length (red triangles), indicating non-overlapping phased piRNA production. Fraction of piRNA pairs was plotted. The 3′-to-5′ end distances between produced piRNAs are plotted. Preferred 1 nt and 25–30 nt distances (red arrows) between LacZ-derived piRNAs indicate that these piRNAs are produced in a phased manner one after the another. (F) Individual piRNAs from a section of the LacZ part of the transcript, which are triggered by NHA-Armi tethering are shown. Only piRNAs with at least a read-count of 10 are plotted.

The *Drosophila* female germline is a widely used model for piRNA research. The fly ovaries are organized into a series of egg chambers, each of which is composed of a single layer of somatic follicle cells that enclose the germline (nurse cells and the developing egg) (Fig 1A). While the germline expresses all three PIWI proteins (Piwi, Aubergine and Ago3), the soma is a simple system operating a primary piRNA pathway that loads Piwi. The *flamenco* cluster is the largest source of piRNAs in this environment, and a fragment consisting of the 1^st^ exon of the *flamenco*, termed piRNA-trigger sequence (PTS), when fused to any heterologous transcript is capable of initiating piRNA biogenesis [9, 13]. We reproduced these results using a reporter consisting of the *flamenco* PTS placed between luciferase and LacZ sequences (Fig 1B). Expression of the reporter in the ovarian somatic cell (OSC) culture model [19] results in the directional production of piRNAs from the downstream LacZ sequences, which are loaded into Piwi (Fig 1B and S1A Fig). Negligible levels of piRNAs are produced in the absence of the PTS element. The same reporter background (two independent constructs), but carrying a perfectly complementary binding site for abundant Piwi-loaded piRNAs (instead of the PTS element) did not result in piRNA production in the OSC system (S1B Fig). Thus we can rule out any role for Piwi slicing in somatic piRNA biogenesis, as already demonstrated [12]. So we hypothesize that the PTS recruits piRNA biogenesis factors to initiate primary processing.

We tested this possibility by directly recruiting piRNA biogenesis factors to a transcript in the ovaries of transgenic flies. To this end, we replaced the PTS sequence in the above reporter with five BoxB (5BoxB) hairpins (Fig 1C). When co-expressed with λ-N peptide-fusion proteins, the BoxB/N-peptide interaction [20] will artificially tether the protein at a central location within the transcript. We first tested Armitage (Armi) which is a highly conserved RNA helicase that is essential for production of all piRNAs in flies [15, 21]. Its orthologue MOV10L1 has a similar role in mice [22–25]. Transgenic fly lines co-expressing both the BoxB reporter and NHA-Armi (with the N-peptide and an HA-tag), specifically in the fly ovarian soma [under control of the *traffic jam*-GAL4 driver (*tj*-GAL4)] were generated. Entry of the reporter into the piRNA pathway was monitored by Piwi immunoprecipitations with fly ovaries and deep sequencing analysis of associated small RNAs (Fig 1A).

Tethering of NHA-Armi triggers piRNA production from the reporter, with most of the reads originating from the BoxB site and the LacZ region downstream (Fig 1C). When HA-Armi (that is unable to tether to the reporter) is expressed, the BoxB reporter produces only low background levels of piRNAs (Fig 1C). Although the reporter sequence has no particular nucleotide bias, the generated artificial piRNAs display a strong bias for having a uridine at the 5′ end (U1-bias), a primary piRNA feature (Fig 1D). Production of piRNAs from the upstream luciferase region is also increased upon the tethering (S1D Fig) but absolute levels remain low. This is not due to any particular features of the sequence, as the same stretch is used for piRNA production in other contexts [9] and as shown below (S5A Fig). However, due to the very low levels of luciferase piRNAs triggered by Armi tethering, we limit the analysis only on piRNAs produced from LacZ region.

To study how the transcript becomes cleaved during primary processing, we calculated the distances between neighbouring piRNAs. When 5′-to-5′ end distances were plotted, we observed peaks at positions ~25, 50 and 75 nt, which correspond to multiples of the approximate length (~24 nt) of a Piwi-bound piRNA (Fig 1E). Measurement of 3′-to-5′ end distances reveals a major peak at position 1 and another one at ~25 nt (Fig 1E). These likely correspond to the distance between 3′ end of a piRNA and the 5′ end of the one immediately downstream (distance of 1 nt) or to the piRNA even further downstream (distance of ~25 nt). These observations indicate a phased/non-overlapping primary piRNA biogenesis mechanism [9–11], where the primary processing machinery moves along the transcript in a stepwise/phased manner to introduce cleavages that simultaneously create the 5′ end of a piRNA and the 3′ end of the preceding one, liberating individual ~24 nt piRNAs (Fig 1F). These phased cleavages are not always precise, but closely spaced (1 nt), giving rise to the additional 5′-to-5′ end distance peak at position 1 (Fig 1E). Note that even in the absence of tethered Armi (when co-expressing HA-Armi), the residual levels of reporter-derived piRNAs generated have a phasing signature (S1C Fig). Taken together, direct binding of Armi to a transcript in the fly ovarian somatic follicle cells identifies it as a primary piRNA precursor, leading to phased piRNA production.

### Armi is a 5′→3′ RNA helicase and mutations that abolish its activity reduce piRNA biogenesis

**Fig 2:**
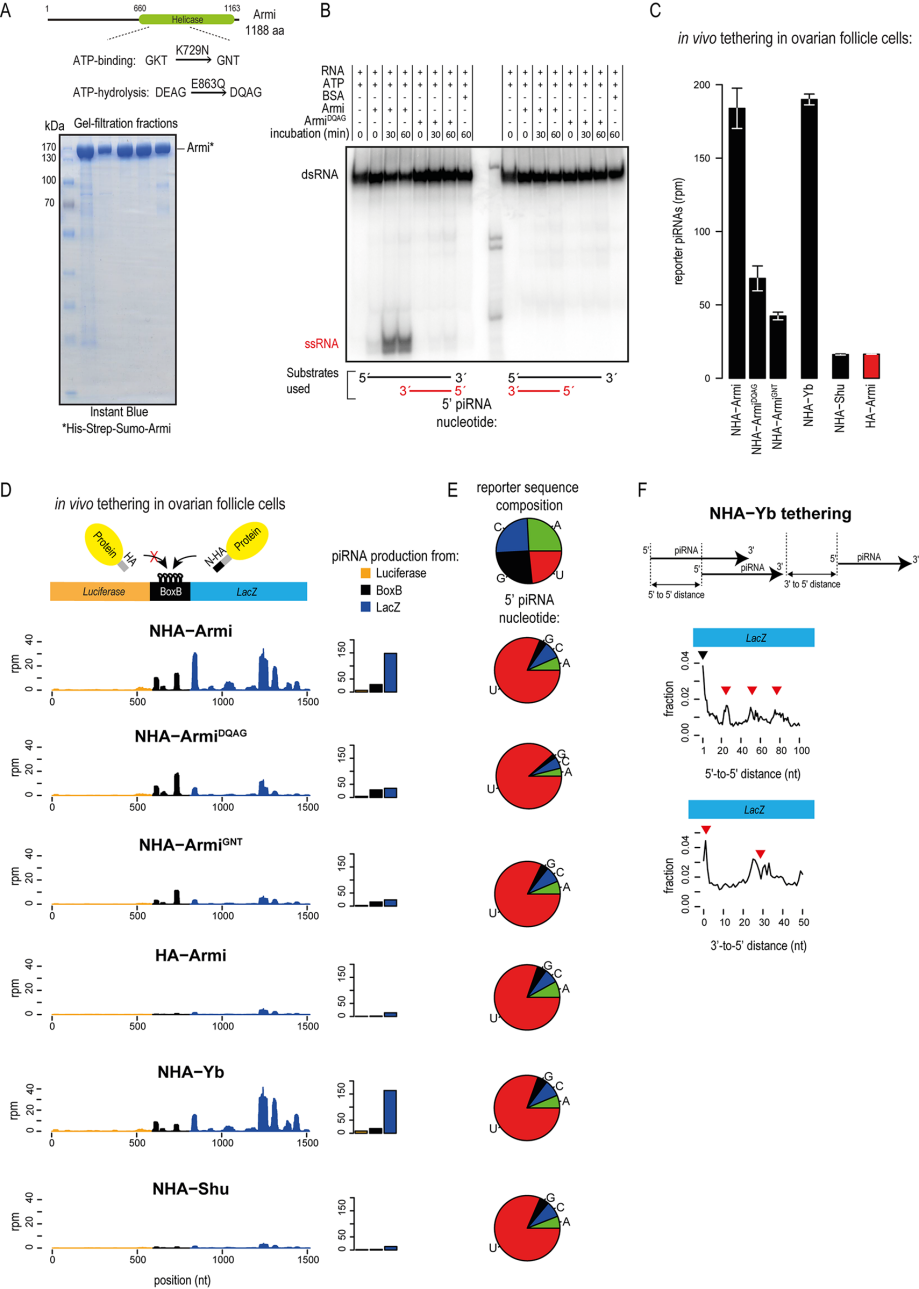
Mutations abolishing the 5′→3′ RNA helicase activity of Armi reduces its ability to trigger piRNA production. (A) Cartoon showing the domain organization of *Drosophila* Armi. Motifs required for ATP-related functions and the mutations made to these are indicated. Quality of purified recombinant Armi protein used in the RNA helicase assay. (B) Recombinant Armi was incubated with RNA duplexes containing different single-stranded overhangs, in the presence or absence of ATP. Only the smaller single-stranded RNA strand (ssRNA) is radioactively labelled at the 5′ end (shown in red). The native polyacrylamide gel shows the ATP-dependent unwinding activity of Armi as revealed by presence of the fast-migrating labelled ssRNA band. The ATPase mutant Armi^DQAG^ is inactive in this assay. (C) Indicated proteins were tethered to the reporter transcript in the somatic follicle cells of transgenic fly ovaries. Average amount of reporter-derived piRNAs produced is plotted with the error bars representing the range of values from two independent experiments. (D) The absolute levels of piRNAs produced are plotted as read coverage (rpm) along the reporter upon tethering of the indicated proteins. The amount of piRNAs produced from different reporter regions is also shown. (E) The 5′ nucleotide composition of produced piRNAs is given. The nucleotide composition of the reporter sequence is shown. (F) When triggered by NHA-Yb, piRNAs generated from the reporter (LacZ part) shows phased primary processing as revealed by the 5′-to-5′ end distance calculations. Peaks (red triangles) at regular intervals correspond to multiples of the average piRNA length of ~25 nt. The black triangle marks the piRNAs starting at neighbouring nucleotides (the distance equal to one). The 3′-to-5′ end distances are plotted. The piRNAs generated tend to be created one after the other in a non-overlapping manner, as demonstrated by the preferred 1 nt distance. The peak at 25–30 nt corresponds to the 5′ end of a piRNA produced further downstream.

Armi is a putative RNA helicase that has conserved sequence motifs essential for ATP binding and ATP hydrolysis (Fig 2A). We directly tested this activity using recombinant *Drosophila* Armi (Fig 2A and S2A Fig). We annealed a 5′-end labelled short single-stranded RNA (ssRNA) with a longer unlabelled complementary sequence to prepare double-stranded RNAs (dsRNAs) with either 5′ or 3′ single-strand overhangs. These RNAs were then incubated with Armi, either in the presence or absence of ATP, and reactions were resolved by 15% native polyacrylamide gel electrophoresis (PAGE). Incubations with Armi, in the presence of ATP, resulted in the appearance of a fast-migrating short ssRNA band, indicative of RNA unwinding activity (Fig 2B and S2B Fig). Interestingly, only the dsRNA with a 5′ single-stranded overhang was used by Armi as a substrate. RNA helicase activity was not observed in the absence of ATP or when the dsRNA has a 3′ single-stranded overhang. Importantly, this activity was abolished when a single amino acid mutation (E863Q) was introduced into the catalytic motif (DEAG→DQAG) of Armi (Fig 2B and S2A Fig). This indicates that Armi is a 5′→3′ RNA helicase and is consistent with the known 5′→3′ RNA helicase activity of its mouse orthologue MOV10L1 [24].

Next, we wished to examine whether the RNA helicase activity is required for tethering-driven piRNA biogenesis. We created transgenic flies co-expressing the NHA-tagged catalytic-dead Armi^DQAG^ mutant protein and the BoxB reporter transcript in the somatic follicle cells of fly ovaries. When tethered to the reporter, overall piRNA production was reduced (2.5-fold) compared to that driven by NHA-Armi (Fig 2C). Examination of piRNA generation across the reporter transcript indicates a dramatic reduction in piRNA levels from transcript, except for those arising from the site of tethering (BoxB sequences) (Fig 2D). A similar reduction (4-fold) in overall piRNA levels is noted when we tethered a second Armi mutant (NHA-Armi^GNT^) that carries a point mutation (K729N) in the ATP binding motif (GKT→GNT) (Fig 2C). Again, piRNA levels decreased across the transcript, except from the site of tethering (Fig 2D). Albeit at reduced levels, piRNAs initiated by Armi helicase mutants display a dominant bias for having a 5′ uridine, indicating genuine primary processing (Fig 2E). In conclusion, helicase activity of Armi is essential for robust piRNA production from the tethered transcript.

### Tethering of Yb, but not of Shutdown, drives piRNA production in the fly ovarian soma

In addition to Armi, other factors are shown to be essential for piRNA biogenesis in the fly ovarian soma. These include the putative RNA helicase Yb [17, 18, 26] and the Hsp90 co-chaperon Shutdown (Shu) [27–29], both of which we tested in our tethering assay using transgenic fly lines. When tethered to the reporter in the fly ovarian somatic follicle cells, Yb led to robust piRNA production from the reporter (Fig 2C). The features of the generated piRNAs mirror that produced by Armi tethering. The sequences have a prominent U1-bias (Fig 2E), and arise in absolute terms mostly from the BoxB sequences and the downstream LacZ region (Fig 2D). Furthermore, measurement of piRNA-end distances reveals that Yb binding triggers phased primary piRNA processing of the transcript (Fig 2F), as demonstrated above for Armi.

In contrast, flies co-expressing NHA-Shu with the reporter revealed only background levels of piRNAs (Fig 2C and 2D). We confirmed by Western analysis that NHA-Shutdown is indeed expressed in fly ovary lysates (S2C Fig). These results indicate that Armi and Yb, but not Shu, when individually tethered to a transcript have the ability to identify it as a primary piRNA precursor in the fly ovarian somatic follicle cells.

### Localization of the tethered factors to Yb bodies promotes piRNA processing in the ovarian soma

**Fig 3:**
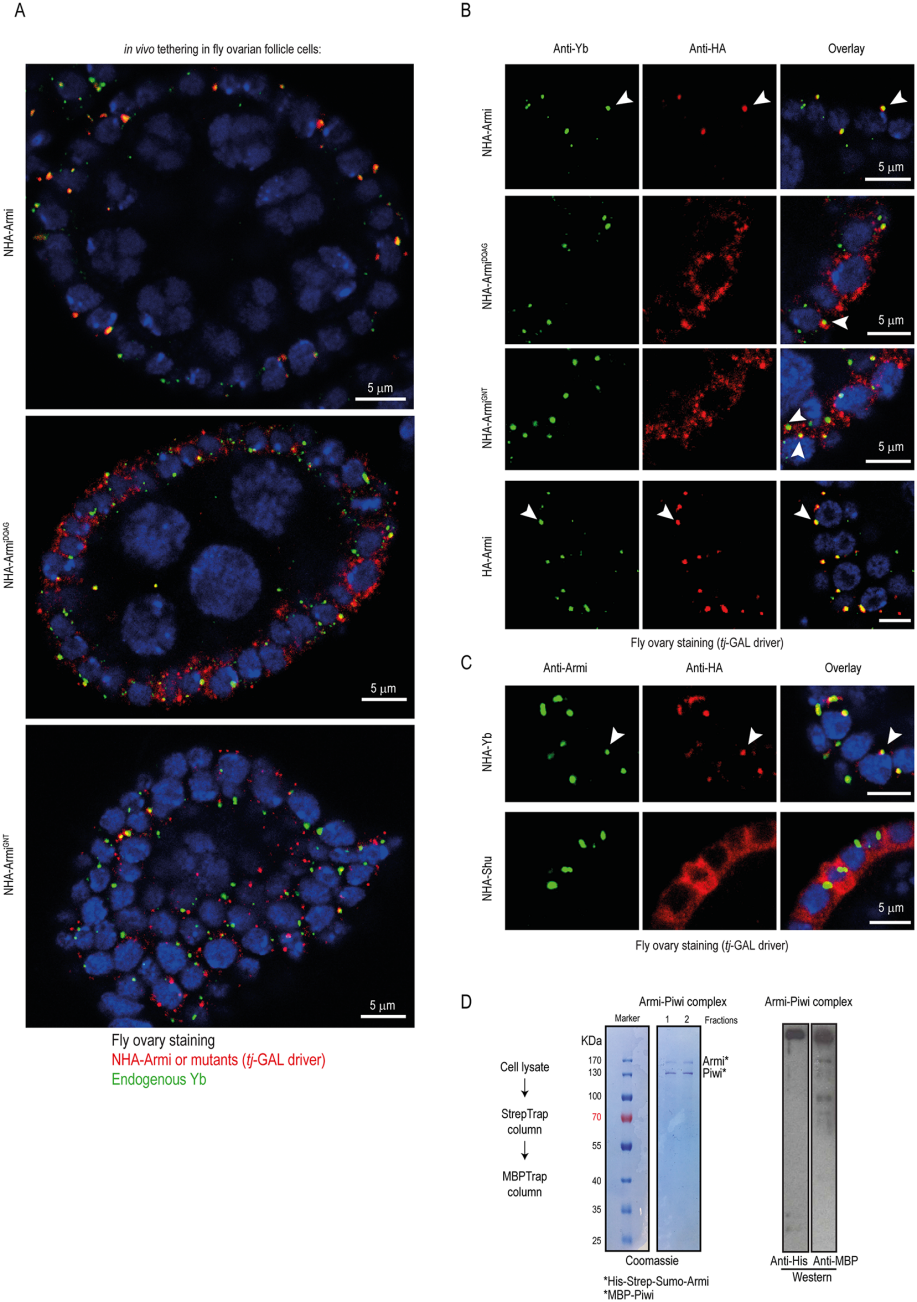
Protein localization in the nuage is a prerequisite for its ability to trigger piRNA biogenesis. (A) Immunofluorescence analysis of NHA-tagged Armi wildtype and mutant versions (red) in transgenic fly ovaries. Single egg chambers are shown, with specific expression (*tj*-GAL4 driver) seen in the somatic follicle cells. Endogenous Yb (green) serves as marker for the cytoplasmic Yb bodies. Wildtype Armi co-localizes in the Yb bodies. Notice that the mutant Armi proteins are dispersed into numerous granules that are not co-stained with Yb. Scale bar is indicated. (B) Zoomed views with Armi or Armi mutant co-localization with endogenous Yb protein indicated (white arrowhead). Scale bars in all the panels correspond to 5μm. (C) NHA-Yb also localizes to the nuage of the ovarian follicle cells as shown by co-localization with endogenous Armi (white arrowhead). In contrast, NHA-Shu is diffused in the cytoplasm. (D) Tagged versions of *Drosophila* Armi and Piwi were co-expressed in insect cell cultures and subjected to a tandem affinity-purification strategy (see S1 Protocols). Coomassie gel shows co-purification of the two proteins, indicating direct interaction. Bands were identified by mass spectrometry and Western blotting to detect the indicated tags.

Most piRNA biogenesis factors are cytoplasmic, where they accumulate in perinuclear granules called nuage [7]. In the fly ovarian somatic follicle cells, this is represented by the Yb body [18, 26]. To examine the localization of the various tethered factors, we carried out anti-HA staining of fly ovaries expressing fusion proteins under control of the soma-specific *tj*-GAL4 driver (Fig 3). Both HA- and NHA-tagged Armi were found in 1–2 granules/cell, which also contain endogenous Yb, identifying their presence within the Yb body (Fig 3A and 3B). This was also true for NHA-Yb, which was co-localized with endogenous Armi (Fig 3C and S2D Fig). In contrast, the Armi^DQAG^ and Armi^GNT^ mutant proteins were more dispersed and accumulated in numerous (up to 10) cytoplasmic granules (Fig 3A and 3B). Although most are non-overlapping with the Yb body, some do overlap (Fig 3B). This mislocalization is not due to any impact on structural integrity of the protein, as the point mutant behaves similar to the wildtype during size-exclusion chromatography (S2A Fig). Thus, loss of RNA helicase activity is directly responsible for failure of the mutants in accumulating in the Yb bodies. Interestingly, NHA-Shu is diffusely present throughout the cytoplasm of ovarian follicle cells, with no co-localization with the Yb body (Fig 3C and S2D Fig). Thus, Armi mutants and Shu that fail to support robust tethering-initiated primary processing are found not to be co-localizing with the Yb body in the fly ovarian follicle cells. It is expected that localization in the Yb body might facilitate association with other piRNA processing factors, for example, like the biochemical association of Armi-Piwi that we demonstrate here (Fig 2D). Taken together, we propose that tethering-induced piRNA production from the reporter transcripts is likely a consequence of the reporter transcript accumulating in the Yb bodies, where it is engaged by the resident piRNA biogenesis machinery.

### Armi and Yb tethering triggers piRNA biogenesis in fly ovarian somatic cell (OSC) cultures

In the above studies, we demonstrated that recruitment of Armi or Yb to a transcript identifies it as a substrate for primary piRNA processing in the fly ovarian somatic follicle cells. To further dissect the requirements from the tethered protein and the reporter RNA for efficient piRNA processing, we made use of the OSC culture system [19], which is a model for the ovarian somatic environment.

**Fig 4:**
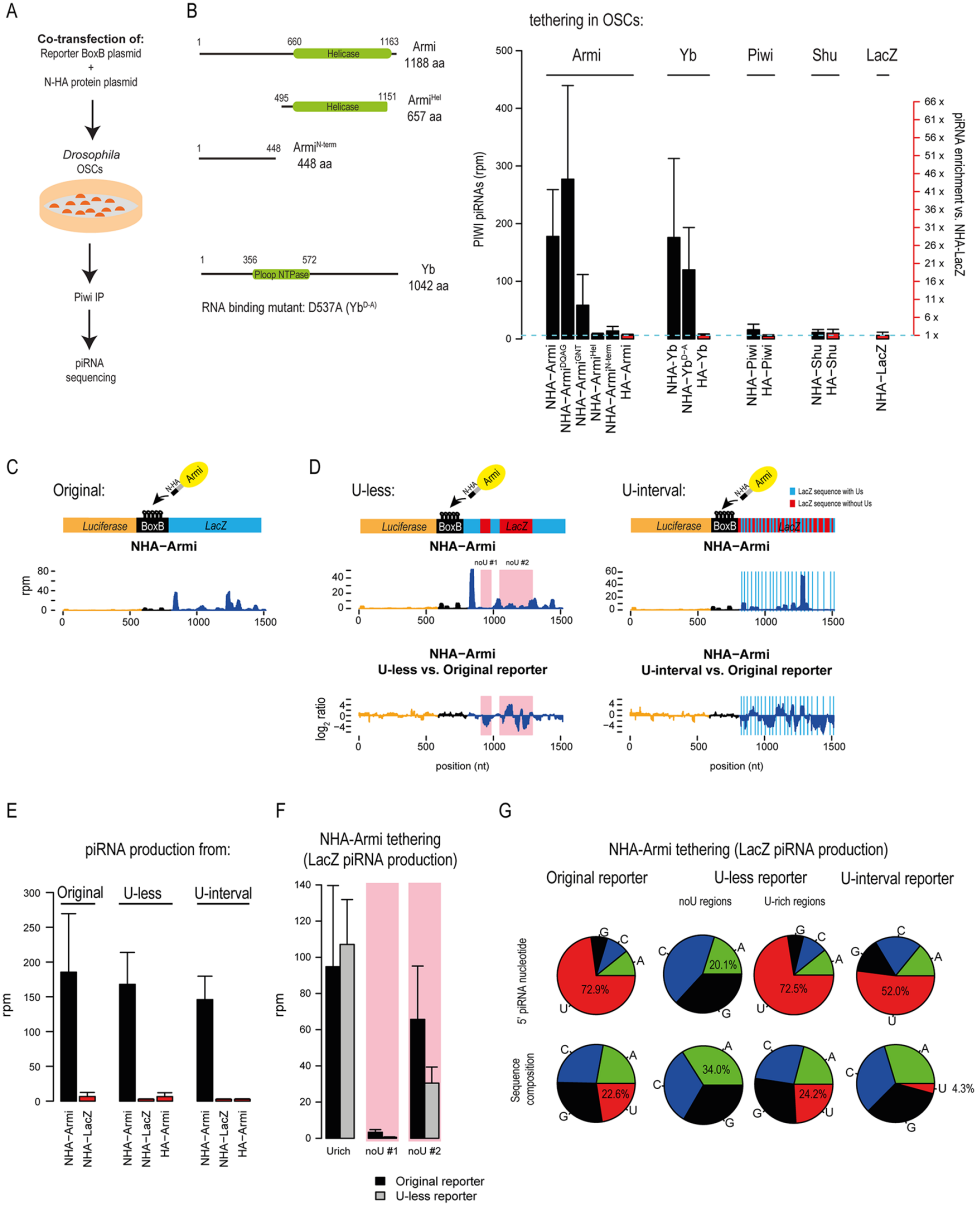
Armi and Yb tethering to a transcript initiates primary processing in OSC system. (A) Ovarian Somatic Cell (OSC) cultures were co-transfected with plasmids expressing fusion proteins and the BoxB reporter. Endogenous Piwi complexes were isolated and associated piRNAs identified by deep sequencing. (B) Various protein constructs used for tethering experiments are shown. Average reporter piRNA production is shown together with the standard deviation from multiple independent transfections. While Armi and Yb tethering initiated piRNA production, tethering of Piwi, Shu and LacZ failed to do so. (C) The absolute levels of NHA-Armi-triggered piRNAs produced from the original reporter are plotted as read coverage (rpm). (D) The original reporter was modified to a U-less reporter that contains two patches that are completely devoid of Us (no-U #1 and no-U #2; red/pink shaded region). U-interval reporter has Us only at specific positions (blue lines), resulting in an overall depletion of uridines in the reporter. Absolute levels of piRNAs produced when tethered with NHA-Armi are plotted. Comparison to piRNA profile of the original reporter shows that changed U compositions strongly affect the distribution of piRNAs from the affected regions. (E) Overall piRNA production from indicated reporters upon tethering with NHA-Armi. Average reporter-derived piRNA production is shown, standard deviation from multiple independent transfections is indicated. (F) The piRNA production from U-less reporter triggered by NHA-Armi is plotted separately for the unmodified LacZ region (U-rich) which is same in both the original and U-less reporters, and for the two patches in the U-less reporter without Us. The regions devoid of Us (no-U #1 and no-U #2) produce less piRNAs than corresponding parts of the original reporter. (G) The frequencies of the 5′ nucleotide in reporter-derived piRNAs are shown along with the nucleotide composition of the source regions. Although the number of Us is strongly decreased in U-interval reporter, the piRNAs still preferentially start at Us. ~30% of these piRNAs start at a specific position with triple Us, however even when these are excluded still ~30% of produced piRNAs contain 5′ U which is much more than expected from the reporter sequence composition (~4%).

OSC cultures were co-transfected with plasmids expressing the BoxB reporter and different NHA- or HA-fusion proteins. Cells were harvested 48-hours post-transfection and libraries were prepared with small RNAs isolated from Piwi immunoprecipitations (Fig 4A and S3A Fig). These experiments largely confirm the findings with the transgenic flies: robust piRNA production when Armi or Yb is tethered to the reporter, but not when tethered with Shu (Fig 4B and 4C and S3B and S3C Fig). Structural integrity of Armi is essential for this functionality, as tethering of the helicase domain alone is unable to trigger piRNA production (Fig 4B and S3C Fig). As shown in fly ovaries, tethering of the Armi^GNT^ mutant resulted in reduced levels of piRNAs, while surprisingly, the catalytic-dead mutant Armi^DQAG^ induced piRNA levels comparable to that seen with the wildtype Armi protein. Interestingly, tethering of Piwi itself did not result in any piRNA production, and behaved similar to tethering of LacZ, a protein unrelated to the piRNA pathway (Fig 4B and S3C Fig). Introduction of a point mutation (D537A) in Yb that is shown to abolish its RNA binding property [26], did not affect its ability to induce piRNA generation (Fig 4B and S3C Fig). This is expected, as artificial tethering to the transcript via N/BoxB system likely negates the requirement for this RNA-binding activity. Finally, we report that we do not see the phasing pattern of piRNA generation in the OSC system (S3D Fig). We have no reason to believe that processing in the OSC proceeds differently than in the fly ovarian somatic follicle cells, so it is likely that technical aspects like transfections and small RNA library quality might have influenced our ability to detect it.

Next, we probed the requirement of uridines in the reporter for tethering-driven piRNA biogenesis, as the generated primary piRNAs display a strong U1-bias (~75%). We modified part of the reporter sequence by creating two patches lacking any Us; no-U#1 and no-U#2 to prepare a U-less reporter and also prepared a U-interval reporter [11] having Us distributed at specific intervals (Fig 4D and S1 Protocols). Lack of uridines in the no-U patches resulted in reduced levels of piRNAs (Fig 4E and 4F and S4 Fig), indicating that Us are preferred, but in the absence of Us any available nucleotide is used for creating 5′ ends of piRNAs. Dramatically, while the U-interval reporter had an overall uridine composition of only ~ 4%, majority of the piRNAs generated displayed a prominent U1-bias (~52%) (Fig 4G and S4E Fig). These results align with the proposed uridine specificity (S4D Fig) of the nuclease Zucchini that generates the piRNA 5′ ends [10, 11]. It is also possible that an additional enrichment of U1-containing piRNAs could be achieved by the nucleotide preference of the MID domain of PIWI proteins [3, 30].

### Armi-initiated processing generates primary piRNAs in the fly ovarian germline

The above studies demonstrate that recruitment of Armi or Yb to a transcript triggers primary piRNA biogenesis that loads Piwi, which is the only PIWI clade member in the fly ovarian somatic follicle cells and the OSC culture system. In contrast, all the three PIWI proteins (Piwi, Aubergine and Ago3) are expressed in the fly germline where there is a dominant dependence on PIWI slicing to initiate piRNA biogenesis. Slicing by Aubergine (Aub) and Ago3 reciprocally loads each other with secondary piRNAs whose 5′ ends are generated by direct slicer action [4, 31]. Additionally, slicer cleavage of a target transcript by Ago3/Aub is also required to load Piwi with a series of phased primary piRNAs [9–11]. So we wished to examine whether our tethering-driven primary piRNA biogenesis might work in the fly germline.

**Fig 5:**
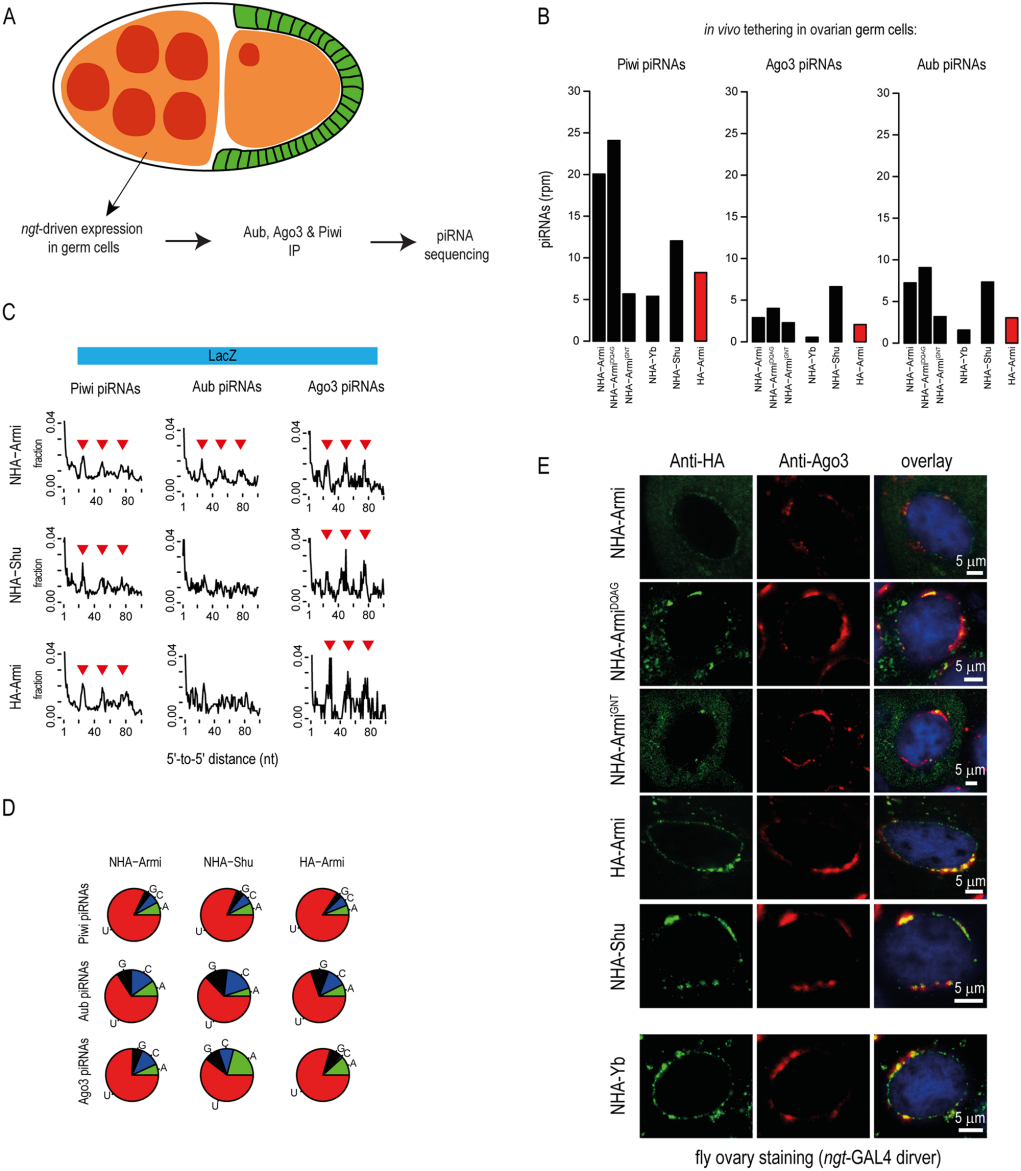
Armi tethering is sufficient to trigger piRNA production in the fly ovarian germ line. (A) A schematic representation of an egg chamber from *Drosophila melanogaster* ovary. The NGT-GAL4 expression system was used to co-express the NHA-tagged proteins with BoxB reporter transcript in the ovarian germ cells. All the three fly PIWI proteins were immunoprecipitated separately and deep sequencing libraries prepared with associated RNAs. (B) Presence of reporter-derived piRNAs in the three PIWI proteins (Aub, Ago3 and Piwi) under conditions where the indicated proteins were co-expressed. Armi and Shu tethering increased the piRNA production when compared to expression of HA-Armi, which cannot bind the reporter transcript. Yb did not induce piRNA production in the ovarian germline. (C) The 5′-to-5′ end-distances of piRNAs were compared for sequences arising from the LacZ region and shows the phased pattern of production. Note that the reporter co-expressed with HA-Armi (which cannot be tethered) also shows lower levels of piRNA production and these piRNAs also show the phasing pattern. (D) The frequencies of the 5′ nucleotide in reporter-derived piRNAs are shown. (E) Immunofluorescence detection of the ectopically expressed proteins in the fly ovarian germ cells. Co-localization of Armi, Shu and Yb with the endogenous Ago3 protein indicates their localization to the nuage.

We created transgenic flies co-expressing the reporter and different fusion proteins in the fly ovarian germline using the NGT-GAL4 driver (Fig 5A). Deep sequencing libraries were prepared with small RNAs present in isolated PIWI complexes (Piwi, Aub and Ago3). When tethered to the reporter, Armi is able to induce piRNA biogenesis that loads all three PIWI proteins, with much more sequences being loaded into Piwi than Aub or Ago3 (Fig 5B and S5 Fig). However, since different polyclonal antibodies are used for PIWI immunoprecipitations, it would be difficult to definitively conclude preferential loading into any protein. The piRNAs associating with the three PIWI proteins display the phasing pattern (Fig 5C) and strong U1-bias (Fig 5D), confirming their generation by primary processing. Interestingly, the Armi^DQAG^ mutant and Shu are also able to trigger piRNA generation. In contrast, the Armi^GNT^ mutant and the soma-specific piRNA factor Yb were inactive in the germline tethering assay (Fig 5B and S5 Fig). Shu tethering induced piRNAs only to a low level compared to that initiated by Armi, but generated piRNAs from the upstream luciferase region also (S5 Fig). Finally, we find a broad correlation between sub-cellular localization of the tethered proteins in the perinuclear nuage (labelled with endogenous Ago3) of the germline nurse cells and their ability to initiate piRNA biogenesis on reporter RNA, with the exception of ectopic Yb, which was also localized in the nuage (Fig 5E). Armi is shown to associate with Ago3, and both proteins accumulate in the nuage along with other piRNA pathway factors [32], allowing entry of the tethered transcripts into piRNA processing machinery. In conclusion, we demonstrate that nuage-localizing factors are able to channel a transcript into primary processing pathway in the fly ovarian germline.

## Discussion

Primary processing is the default pathway that generates piRNAs in all animal germlines. Since precursors are not unlike other cellular mRNAs or non-coding transcripts, there should be mechanisms in place to specify their entry into the processing machinery. Much is known about the secondary processing pathway operating in the fly ovarian germline, where PIWI slicing of a target transcript results in its entry into piRNA processing [9–11]. However, this depends on pre-existing piRNAs, which are suggested to be provided by maternal deposition in the egg. In contrast, primary processing has to kick-start piRNA production in the absence of pre-existing piRNAs (as in fly ovarian soma), and without the use of PIWI slicing [12]. How this is achieved is poorly understood.

Previous work implicated a role for sequences at the 5′ end of precursors termed piRNA-trigger sequences (PTSs) in recruiting the primary processing machinery in the OSC culture model [9, 13]. PTS elements are poorly defined and lack conservation, preventing their detailed study, but our work provides strong support to the hypothesis that they might provide landing sites for specific piRNA biogenesis factors.

In this study, we demonstrate that presence of a perfectly complementary site for abundant piRNAs within a reporter did not trigger piRNA biogenesis in the OSC system (S1B Fig). Instead, we show that artificial recruitment of primary biogenesis factors, Armi and Yb, to a reporter transcript is sufficient to identify it as a primary piRNA precursor (Figs 1 and 2). Among these, Armi is highly conserved and works in all the systems tested: fly ovarian soma and germline, and in the OSC cultures. Armi [14, 15, 21] and its mouse orthologue MOV10L1 [22–25] are absolutely essential for biogenesis of all piRNAs in flies and mice. In contrast, Yb is restricted to *Drosophila*, pointing to a non-conserved role for the protein in the fly somatic follicle cells [16]. The known interaction between Yb and Armi [16, 17] might ensure that Yb-tethered transcripts enter primary processing in the fly soma and in the OSC system (Figs 2 and 4), while lack of functionality of ectopically expressed Yb in the germline (Fig 5) could be due to competition from its germ cell specific homologues BoYb and SoYb [16].

Armi- or Yb-mediated primary processing of the tethered transcript strongly resembles that initiated by PIWI slicing in the fly germline [9–11] or in the mouse male germline [33, 34]. In both situations the transcript undergoes phased processing to generate piRNAs with a strong U1-bias, and predominantly proceeds in a 5′→3′ direction. This points to different modes of precursor identification that eventually channels the transcript into a common piRNA biogenesis machinery. We propose that tethering by nuage- or Yb body-localizing factors results in a fast-track access for the transcript to the resident piRNA biogenesis machinery in these cytoplasmic processing sites. RNA helicases are shown to recognize target RNAs in a sequence-independent manner [35], and this raises the possibility that any spurious association of piRNA biogenesis factors with other cellular RNAs can lead to their entry into the piRNA pathway, a situation that germ cells must actively prevent from happening. We believe that our tethering-mediated piRNA biogenesis strategy provides a valuable tool for further exploring the molecular mechanisms of primary piRNA processing and may even be harnessed for creation of designer small RNAs that can target any germline gene for epigenetic silencing.

## Materials and methods

### Antibodies used in this study

Antibodies to all three *Drosophila* PIWI proteins used in this study were previously described [9]. These include rabbit polyclonal antibodies (two rabbits: GJKO and GJLD) to *Drosophila* Piwi that were generated (EMBL Protein expression and purification core facility) against an insoluble antigen (Piwi antigen: 42–178 aa) produced in *E*.*coli* as an inclusion body. Single rabbits were used to generate the antibodies to *Drosophila* Aub and Ago3 (Aub antigen: 1–200 aa; Ago3 antigen:1–200 aa). Immunized rabbit sera were directly used for immunoprecipitation.

### Constructs for OSC experiments

For expression in the *Drosophila* ovarian somatic cell (OSC) cultures [19], we used the pAC5.1 vector (Life Technologies) driving expression from the fly *actin* promoter [9]. For expression of either HA-tag (pAC-HA) or N-HA-tag fusions (pAC-NHA), the pAC5.1 vector was further modified to add the necessary coding sequences. The HA tag is for detection of the expressed protein and the λN-peptide is for artificially tethering the fusion protein to a transcript containing BoxB sequences [20].

### Constructs and crosses for transgenic *Drosophila* experiments

For creating transgenic fly lines, the coding sequences for NHA- or HA-tagged fusions of Armi, Yb or Shu, and the point mutant versions were inserted into the pUASp_attB_delK10 plasmid containing the *white+* gene marker. These were used for site-specific integration (BestGene, Inc) in the *Drosophila* genome using the PhiC31 (ΦC31) integrase-mediated transgenesis system. Details of crosses are given in S1 Protocols.

### OSC cell culture and electroporation

*Drosophila* ovarian somatic cell (OSC; gift of Dr. M. Siomi, University of Tokyo) culture system is representative of the fly ovarian somatic follicle cells [19]. OSCs were cultured in 75 cm flasks and grown to 80% confluence. Approximately 3.5x10^6^ cells were used for each electroporation reaction using Cell Line Nucleofector Kit V (Lonza, Cat No. VCA-1003) and were plated in 6-well plate. Further details in S1 Protocols.

### Purification of recombinant Armi or Armi-Piwi complex

For production of recombinant proteins in the insect cells the following ovary-derived cells were used: Sf21 or Sf9 from Fall Army worm *Spodoptera frugiperda* or High Five (Hi5) from the cabbage looper, *Trichoplusia ni*. Expression of desired coding sequences was carried out with the use of recombinant Baculoviruses. Either single or multiple coding sequences were integrated into the Baculovirus genome using the MultiBac protein expression system [36]. The coding sequence for *Drosophila* Armitage (Armi) was isolated by RT-PCR from fly ovarian total RNA, while the codon-optimized DNA sequence was commercially synthesized (Shanghai ShineGene Molecular Biotech,Inc.). Detailed purification steps in S1 Protocols.

### RNA unwinding assay

RNA unwinding reaction was performed as described [37, 38] with some modifications. Single stranded RNA oligos were chemically synthesized (Microsynth, CH) and sequences are given in S1 Protocols. Substrates for RNA unwinding assay were prepared by annealing a 5′-endlabelled strand that was annealed with its unlabelled complementary partner. For details see S1 Protocols.

### Bioinformatic analysis of small RNA libraries

Reads were sorted into individual libraries based on the barcodes and the 3′ adapter sequences were clipped using cutadapt (DOI:http://dx.doi.org/10.14806/ej.17.1.200). Reads which are at least 15 nucleotides in length were used for subsequent analysis and the independent replicated libraries were merged together. Reads were then aligned to the desired reporter sequence using bowtie [39] allowing no mismatches. Analyses were performed as previously described [9]. See S1 Protocols for details.

## Supporting information

S1 Fig Perfectly complementary sites for piRNAs in a reporter does not trigger piRNA production in OSC cultures. (A) The length profile of piRNAs produced from the LacZ part of the reporter, containing the *flam* piRNA trigger sequence (PTS), when transfected into OSC cultures. Comparison of 5′-to-5′ and 3′-to-5′ end-distances between produced piRNAs does not show any regular positioning of the piRNAs. Many of the piRNAs are overlapping and start close to each other with most pairs starting at neighbouring nucleotides (5′-to-5′ distance equal to one). (B) Presence of a perfectly complementary binding site for abundant Piwi-bound piRNAs in a reporter does not initiate piRNA production in OSC cultures. The piRNA production is shown along the reporter as the read coverage (rpm) together with the coverage of targeting piRNAs. Two independent reporters with a single binding site for different piRNAs (piRNA 1 and piRNA 2) were tested. (C) Co-expression of the BoxB reporter with HA-Armi (which cannot bind the reporter) in follicle cells of transgenic fly ovaries. Very low levels of piRNAs are nevertheless produced in these conditions and comparison of 5′-to-5′ and 3′-to-5′ end distances between produced piRNAs reveal phased pattern of production. This is indicated by enriched 3′-to-5′ distance equal to one and 5′-to-5′ distance peaks which are separated by distances equal to piRNA length. (D) To assess the effect of Armi tethering on piRNA production, the piRNA reporter coverage of NHA-Armi was compared to HA-Armi control, which does not bind the reporter transcript. The log_2_ changes in piRNA coverage are shown. Overall change of piRNA levels for individual parts of the transcript is also provided.
(TIF)

S2 Fig Recombinant Armi purification and RNA helicase assays. (A) Recombinant Armi and Armi^DQAG^ were expressed and purified from insect cells. Gel filtration factions of indicated proteins were resolved by SDS-PAGE and stained with Instant Blue stain. Both wildtype and mutant Armi proteins elute at similar fractions, indicating no gross change in structural integrity of the protein due to the mutation. (B) ATPase assay with recombinant Armi. RNA duplexes were prepared using a short 5′-end labelled single-stranded RNA (ssRNA) that is annealed to its longer complementary strand such that duplexes have either 5′ or 3′ overhangs. The native polyacrylamide gel is shown resolving the double-stranded RNAs (dsRNAs) and ssRNAs. Duplexes with a 5′ single-stranded overhang is used as a substrate by Armi, as indicated by the fast-migrating ssRNA band. Heat-denaturation also releases this ssRNA band. (C) Western analysis of HA-tagged proteins in fly ovary lysates. Proteins were expressed with the *tj*-GAL4 driver in the fly ovarian soma. Both NHA-Yb and NHA-Shu are expressed. Tubulin is used as loading control. (D) Immunofluorescence detection of indicated HA-tagged proteins (red) and co-localization with an endogenous Yb body marker protein (green).
(TIF)

S3 Fig Tethering Armi and Yb to a transcript triggers piRNA production in the *Drosophila* ovarian somatic cell (OSC) culture model. (A) A BoxB reporter was tethered with indicated proteins in *Drosophila* OSC cultures and generated piRNAs analysed by deep sequencing Piwi-bound RNAs. Length profiles of reporter-derived piRNAs are shown. The profiles of piRNAs derived from luciferase and LacZ regions are plotted separately. Note that LacZ region produces substantially more piRNAs in absolute terms (see panel C). (B) The BoxB reporter transcript alone produces low background levels of piRNAs when expressed in OSCs. (C) The absolute levels of piRNAs produced from the reporter are plotted as read coverage (rpm). The protein co-expressed with the reporter is indicated. The amount of piRNAs produced from separate reporter regions is also shown. (D) The 5′-to-5′ end and 3′-to-5′ distances between the piRNAs are shown for the LacZ region of the reporter. No preferred distances between piRNAs can be observed except for the preference of the 5′ ends to start at neighbouring nucleotides (5′-to-5′ distance equal to one). This situation is different from the phasing observed with the somatic follicle cells in the fly ovary.
(TIF)

S4 Fig Distribution of piRNAs is affected by frequency and position of uridines in a precursor transcript in the OSC culture system. (A) Three different BoxB reporters (Original, U-less or U-interval) were tethered with NHA-Armi and piRNA production examined. Length profiles of reporter-derived piRNAs are shown. (B) The distribution of 5′ end of piRNAs is shown along the reporter for the U-less and U-interval reporters. U-less reporter was designed to contain two regions that are completely devoid of Us (no-U #1 and no-U #2; pink shaded regions). U-interval reporter has Us at specific positions (blue lines). Comparison of these 5′ end distributions with the original reporter shows striking influence of U distribution on the distribution of 5′ piRNA ends. (C) Mutual comparison of piRNA coverage along the part of LacZ region for the original, U-less and U-interval reporters upon tethering of NHA-Armi. The U-less and U-interval reporters have similar coverage in the affected region which is completely devoid of Us (in case of the U-less reporter) or strongly depleted of Us (in case of the U-interval reporter). (D) Nucleotide composition is shown for the nucleotide immediately following the 3′ end of the piRNAs. LacZ piRNAs triggered by NHA-Armi tethering were analysed. The dominance of Us suggests a sequential piRNA biogenesis mechanism that simultaneously generates the (U1) 5′ end of a piRNA and 3′ end of the preceding one. Nevertheless, we were unable to compute the phasing pattern as mentioned in S3D Fig. (E) Only part of the LacZ sequence is shown for the different reporters. Individual piRNAs produced from the original, U-less and U-interval reporters are plotted. Only sequences that were sequenced at least 10 times were considered. The pink shaded region defines the region which is completely devoid of Us in the U-less reporter. Note that even in the absence of Us, primary piRNA processing continues in a phased manner from the unchanged part of the reporter (upstream), into the no-U region, and then into the downstream regions.
(TIF)

S5 Fig Armi tethering drives piRNA production from a BoxB reporter in fly ovarian germ cells. A BoxB reporter was tethered with indicated proteins (NHA-tagged) in the germ cells of fly ovaries, and piRNAs generated from the reporter were detected by immunoprecipitations of the three PIWI proteins. Note that HA-Armi is unable to tether to the reporter, so serves as the background control. (A) The absolute piRNA coverages are shown. Note that the scales are different when plotting the absolute piRNA levels. Ago3, Aub and Piwi piRNA distribution was analysed separately. (B) Absolute piRNA levels produced from the different regions (luciferase, BoxB and LacZ) of the reporter, when tethered by indicated proteins, are shown. Note that NHA-Shutdown (Shu) tethering results in piRNA generation from the entire transcript (including the luciferase part).
(TIF)

S1 Table List of all deep sequencing libraries used in this study. All deep sequencing libraries generated in this study are deposited with Gene Expression Omnibus (GEO) under the accession numbers GSE102013.
(PDF)

S1 Protocols Detailed protocols used in this study. A detailed description of protocols, materials and reporter sequences used in this study is given.
(DOCX)
