# Supplementary figures and images for: Recruitment of Armitage and Yb to a transcript triggers its phased processing into primary piRNAs in Drosophila ovaries
Source: PLoS Genet. 2017 Aug 21;13(8):e1006956. doi: 10.1371/journal.pgen.1006956 (PMC5578672; doi:10.1371/journal.pgen.1006956)

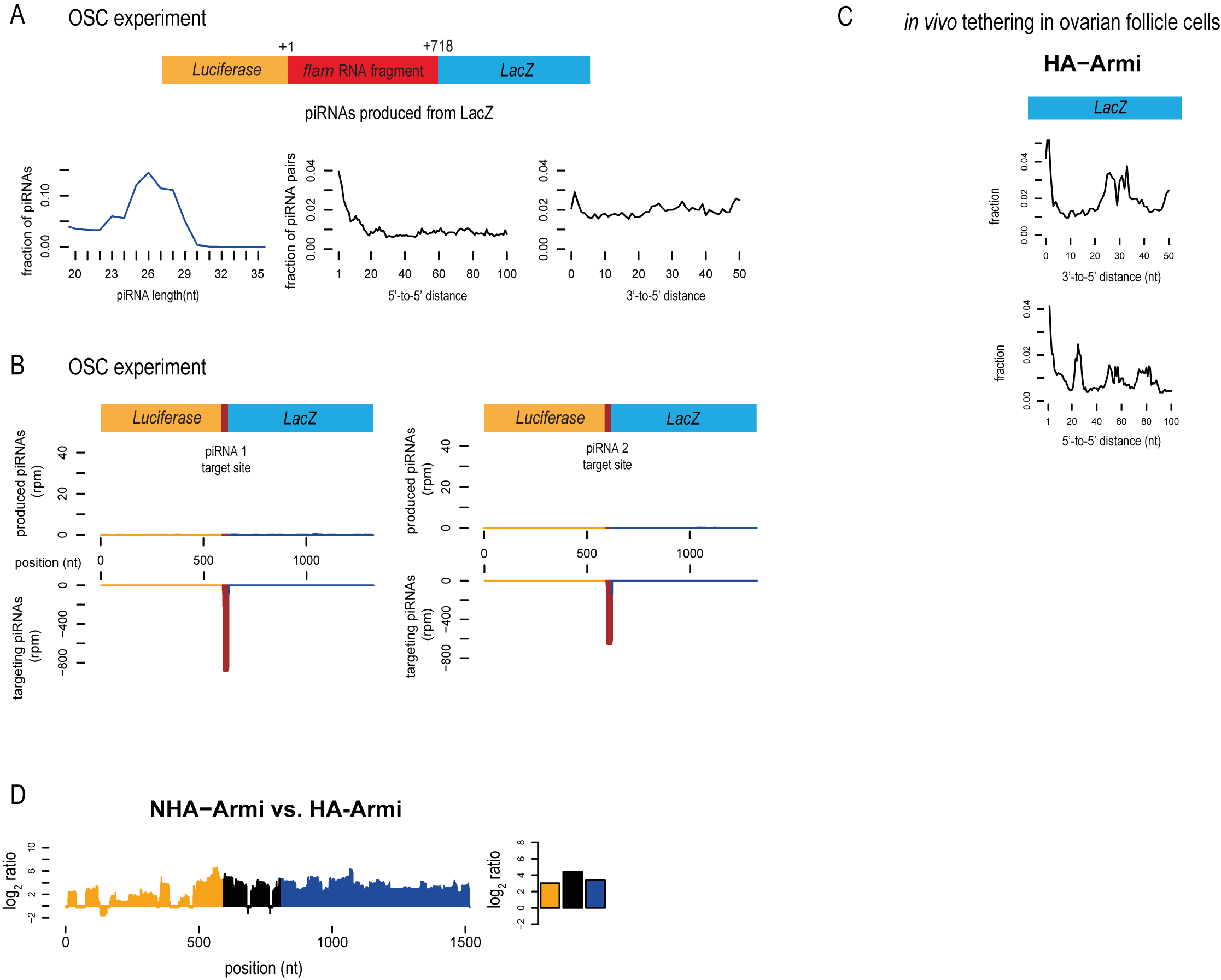

Supplement: S1 Fig — (A) The length profile of piRNAs produced from the LacZ part of the reporter, containing the flam piRNA trigger sequence (PTS), when transfected into OSC cultures. Comparison of 5′-to-5′ and 3′-to-5′ end-distances between produced piRNAs does not show any regular positioning of the piRNAs. Many of the piRNAs are overlapping and start close to each other with most pairs starting at neighbouring nucleotides (5′-to-5′ distance equal to one). (B) Presence of a perfectly complementary binding site for abundant Piwi-bound piRNAs in a reporter does not initiate piRNA production in OSC cultures. The piRNA production is shown along the reporter as the read coverage (rpm) together with the coverage of targeting piRNAs. Two independent reporters with a single binding site for different piRNAs (piRNA 1 and piRNA 2) were tested. (C) Co-expression of the BoxB reporter with HA-Armi (which cannot bind the reporter) in follicle cells of transgenic fly ovaries. Very low levels of piRNAs are nevertheless produced in these conditions and comparison of 5′-to-5′ and 3′-to-5′ end distances between produced piRNAs reveal phased pattern of production. This is indicated by enriched 3′-to-5′ distance equal to one and 5′-to-5′ distance peaks which are separated by distances equal to piRNA length. (D) To assess the effect of Armi tethering on piRNA production, the piRNA reporter coverage of NHA-Armi was compared to HA-Armi control, which does not bind the reporter transcript. The log2 changes in piRNA coverage are shown. Overall change of piRNA levels for individual parts of the transcript is also provided. (TIF) [file pgen.1006956.s001.tif]

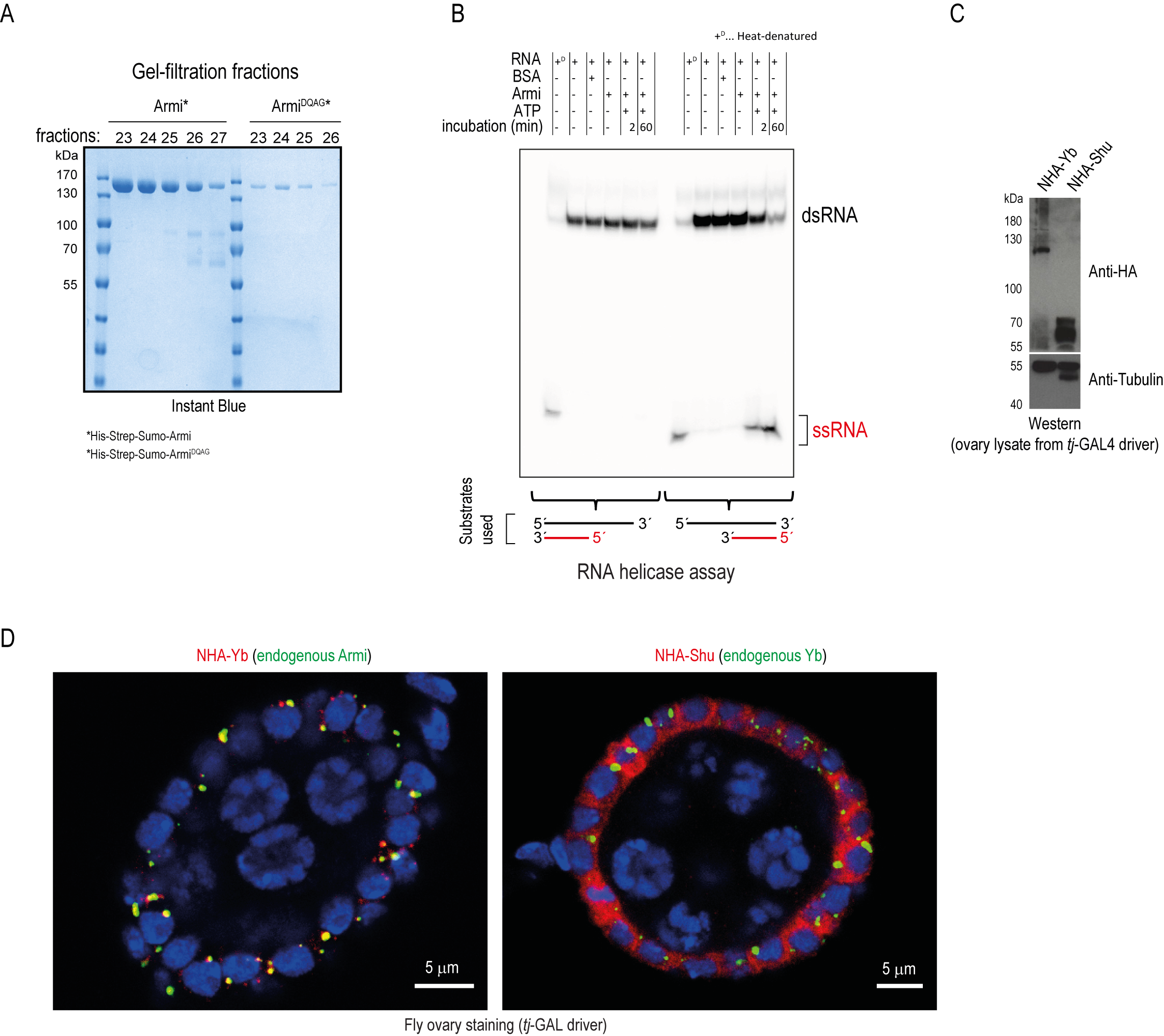

Supplement: S2 Fig — (A) Recombinant Armi and ArmiDQAG were expressed and purified from insect cells. Gel filtration factions of indicated proteins were resolved by SDS-PAGE and stained with Instant Blue stain. Both wildtype and mutant Armi proteins elute at similar fractions, indicating no gross change in structural integrity of the protein due to the mutation. (B) ATPase assay with recombinant Armi. RNA duplexes were prepared using a short 5′-end labelled single-stranded RNA (ssRNA) that is annealed to its longer complementary strand such that duplexes have either 5′ or 3′ overhangs. The native polyacrylamide gel is shown resolving the double-stranded RNAs (dsRNAs) and ssRNAs. Duplexes with a 5′ single-stranded overhang is used as a substrate by Armi, as indicated by the fast-migrating ssRNA band. Heat-denaturation also releases this ssRNA band. (C) Western analysis of HA-tagged proteins in fly ovary lysates. Proteins were expressed with the tj-GAL4 driver in the fly ovarian soma. Both NHA-Yb and NHA-Shu are expressed. Tubulin is used as loading control. (D) Immunofluorescence detection of indicated HA-tagged proteins (red) and co-localization with an endogenous Yb body marker protein (green). (TIF) [file pgen.1006956.s002.tif]

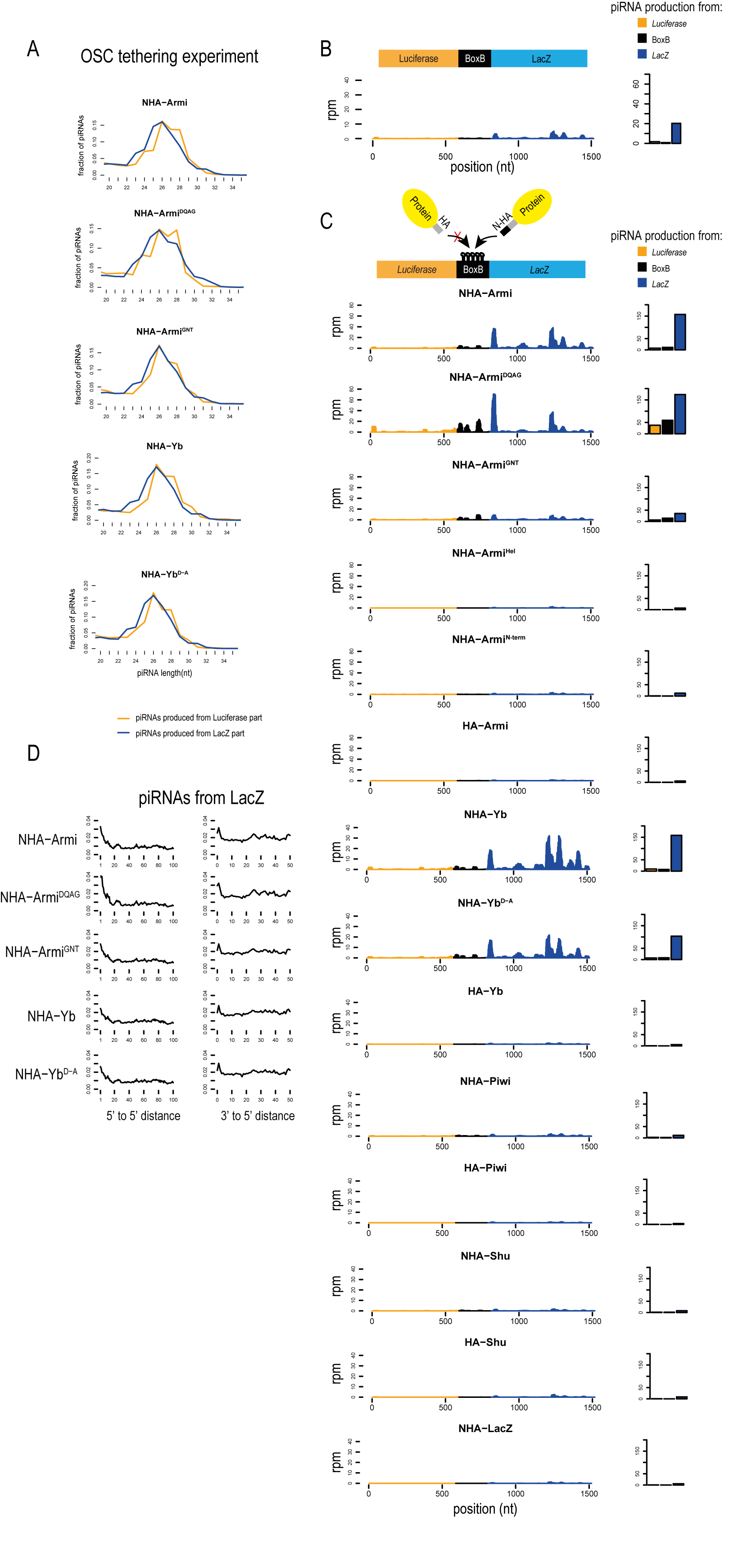

Supplement: S3 Fig — (A) A BoxB reporter was tethered with indicated proteins in Drosophila OSC cultures and generated piRNAs analysed by deep sequencing Piwi-bound RNAs. Length profiles of reporter-derived piRNAs are shown. The profiles of piRNAs derived from luciferase and LacZ regions are plotted separately. Note that LacZ region produces substantially more piRNAs in absolute terms (see panel C). (B) The BoxB reporter transcript alone produces low background levels of piRNAs when expressed in OSCs. (C) The absolute levels of piRNAs produced from the reporter are plotted as read coverage (rpm). The protein co-expressed with the reporter is indicated. The amount of piRNAs produced from separate reporter regions is also shown. (D) The 5′-to-5′ end and 3′-to-5′ distances between the piRNAs are shown for the LacZ region of the reporter. No preferred distances between piRNAs can be observed except for the preference of the 5′ ends to start at neighbouring nucleotides (5′-to-5′ distance equal to one). This situation is different from the phasing observed with the somatic follicle cells in the fly ovary. (TIF) [file pgen.1006956.s003.tif]

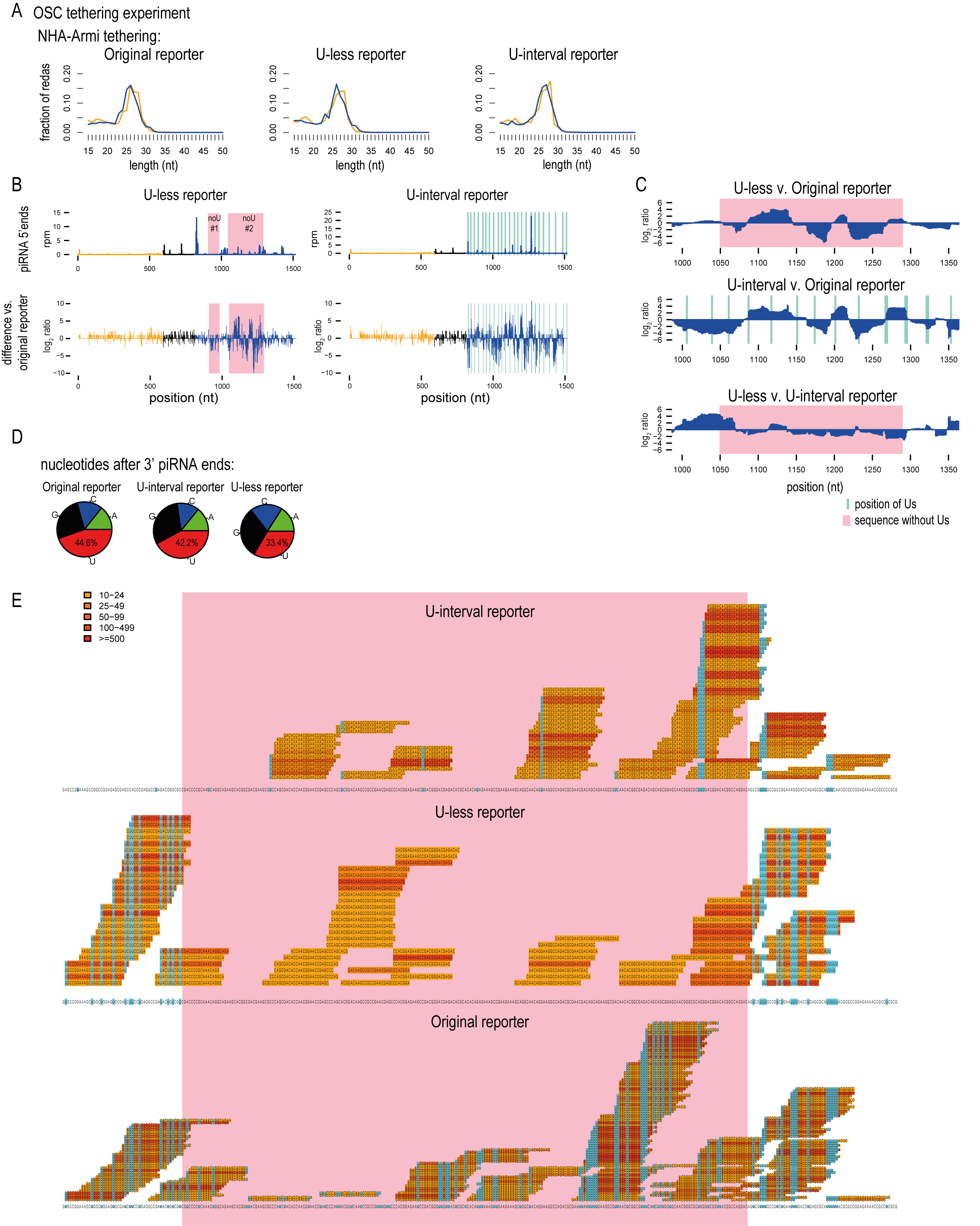

Supplement: S4 Fig — (A) Three different BoxB reporters (Original, U-less or U-interval) were tethered with NHA-Armi and piRNA production examined. Length profiles of reporter-derived piRNAs are shown. (B) The distribution of 5′ end of piRNAs is shown along the reporter for the U-less and U-interval reporters. U-less reporter was designed to contain two regions that are completely devoid of Us (no-U #1 and no-U #2; pink shaded regions). U-interval reporter has Us at specific positions (blue lines). Comparison of these 5′ end distributions with the original reporter shows striking influence of U distribution on the distribution of 5′ piRNA ends. (C) Mutual comparison of piRNA coverage along the part of LacZ region for the original, U-less and U-interval reporters upon tethering of NHA-Armi. The U-less and U-interval reporters have similar coverage in the affected region which is completely devoid of Us (in case of the U-less reporter) or strongly depleted of Us (in case of the U-interval reporter). (D) Nucleotide composition is shown for the nucleotide immediately following the 3′ end of the piRNAs. LacZ piRNAs triggered by NHA-Armi tethering were analysed. The dominance of Us suggests a sequential piRNA biogenesis mechanism that simultaneously generates the (U1) 5′ end of a piRNA and 3′ end of the preceding one. Nevertheless, we were unable to compute the phasing pattern as mentioned in S3D Fig. (E) Only part of the LacZ sequence is shown for the different reporters. Individual piRNAs produced from the original, U-less and U-interval reporters are plotted. Only sequences that were sequenced at least 10 times were considered. The pink shaded region defines the region which is completely devoid of Us in the U-less reporter. Note that even in the absence of Us, primary piRNA processing continues in a phased manner from the unchanged part of the reporter (upstream), into the no-U region, and then into the downstream regions. (TIF) [file pgen.1006956.s004.tif]

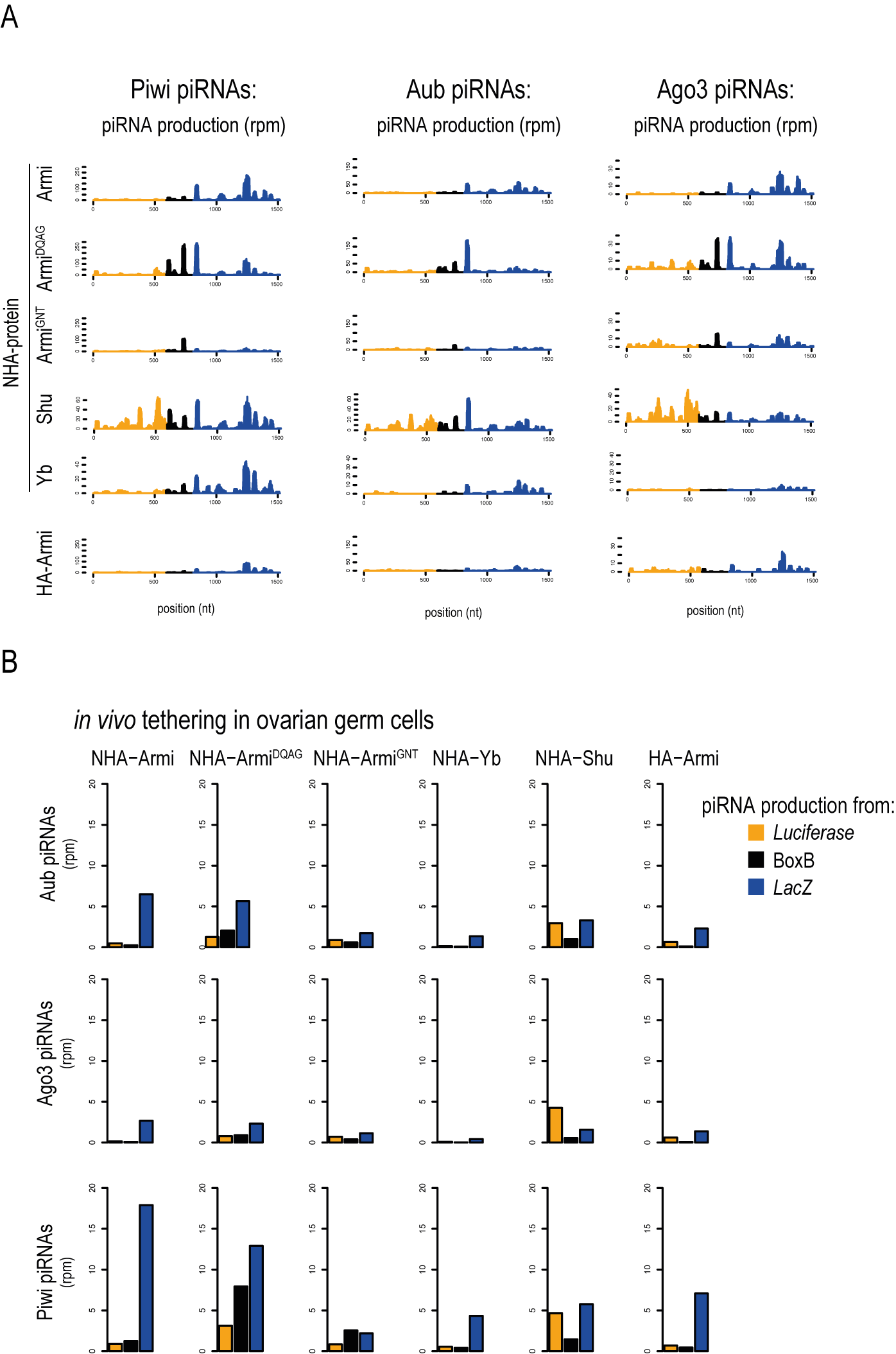

Supplement: S5 Fig — A BoxB reporter was tethered with indicated proteins (NHA-tagged) in the germ cells of fly ovaries, and piRNAs generated from the reporter were detected by immunoprecipitations of the three PIWI proteins. Note that HA-Armi is unable to tether to the reporter, so serves as the background control. (A) The absolute piRNA coverages are shown. Note that the scales are different when plotting the absolute piRNA levels. Ago3, Aub and Piwi piRNA distribution was analysed separately. (B) Absolute piRNA levels produced from the different regions (luciferase, BoxB and LacZ) of the reporter, when tethered by indicated proteins, are shown. Note that NHA-Shutdown (Shu) tethering results in piRNA generation from the entire transcript (including the luciferase part). (TIF) [file pgen.1006956.s005.tif]
