# Supplementary material for: Recruitment of Armitage and Yb to a transcript triggers its phased processing into primary piRNAs in Drosophila ovaries
Source: PLoS Genet. 2017 Aug 21;13(8):e1006956. doi: 10.1371/journal.pgen.1006956 (PMC5578672; doi:10.1371/journal.pgen.1006956)
Supplement: S1 Table — All deep sequencing libraries generated in this study are deposited with Gene Expression Omnibus (GEO) under the accession numbers GSE102013. (PDF) [file pgen.1006956.s006.pdf]

#	sample	expressed reporter	expressed protein	IP	description	read counts	after adaptor clipping	reporter piRNAs (24-30nt)	expressed in
1	RR242	1347	none	PIWI	reporter: Luc-LacZ	45400153	31755210	453	OSCs
2	RR243	1347	none	PIWI	reporter: Luc-LacZ	49817214	34905687	271	OSCs
7	RR248	1347	none	PIWI	reporter: Luc-LacZ	45818946	41476378	185	OSCs
8	RR249	1347	none	PIWI	reporter: Luc-LacZ	53235705	38526657	150	OSCs
3	RR244	1355	none	PIWI	reporter: Luc-Flam718-LacZ	47400545	34136474	6633	OSCs
4	RR245	1355	none	PIWI	reporter: Luc-Flam718-LacZ	46484415	31535069	5078	OSCs
5	RR246	1356	none	PIWI	reporter: Luc-5BoxB-LacZ	41848429	28960174	1076	OSCs
6	RR247	1356	none	PIWI	reporter: Luc-5BoxB-LacZ	36707915	26165794	733	OSCs
9	RR250	1357	none	PIWI	reporter: Luc-piRNA1-LacZ	50189193	37676526	64	OSCs
10	RR251	1357	none	PIWI	reporter: Luc-piRNA1-LacZ	50008169	29884732	32	OSCs
11	RR252	1358	none	PIWI	reporter: Luc-piRNA2-LacZ	40555355	32489818	35	OSCs
12	RR253	1358	none	PIWI	reporter: Luc-piRNA2-LacZ	42474961	31871279	46	OSCs
13	RR259	1356	NHA-Armi	PIWI	NHA-Armi+reporter	51911787	36331923	6556	OSCs
14	RR269	1356	NHA-Armi	PIWI	NHA-Armi+reporter	48188326	30679254	5796	OSCs
15	RR285	1356	NHA-Armi	PIWI	NHA-Armi+reporter	48866713	38296889	15543	OSCs
16	RR310	1356	NHA-Armi	PIWI	NHA-Armi+reporter	18275170	12804800	3174	OSCs
17	RR320	1356	NHA-Armi	PIWI	NHA-Armi+reporter	35245580	24856197	5307	OSCs
18	RR260	1356	NHA-Armi-GNT	PIWI	NHA-Armi-GNT+reporter	53346763	38587783	7991	OSCs
19	RR270	1356	NHA-Armi-GNT	PIWI	NHA-Armi-GNT+reporter	45005773	28773416	716	OSCs
20	RR286	1356	NHA-Armi-GNT	PIWI	NHA-Armi-GNT+reporter	46649735	37398610	2330	OSCs
21	RR314	1356	NHA-Armi-GNT	PIWI	NHA-Armi-GNT+reporter	22061028	14948195	567	OSCs
22	RR324	1356	NHA-Armi-GNT	PIWI	NHA-Armi-GNT+reporter	31514105	23179180	1597	OSCs
23	RR315	1356	NHA-ArmiDQ	PIWI	NHA-ArmiDQ+reporter	20256206	14329555	7940	OSCs
24	RR325	1356	NHA-ArmiDQ	PIWI	NHA-ArmiDQ+reporter	32931433	22228119	5346	OSCs
25	RR311	1356	NHA-ArmiDel1	PIWI	NHA-ArmiDel1+reporter	19060690	13628393	178	OSCs
26	RR321	1356	NHA-ArmiDel1	PIWI	NHA-ArmiDel1+reporter	30129621	22798882	205	OSCs
27	RR313	1356	NHA-ArmiDel3	PIWI	NHA-ArmiDel3+reporter	20010239	15078465	388	OSCs
28	RR323	1356	NHA-ArmiDel3	PIWI	NHA-ArmiDel3+reporter	31450044	23754487	259	OSCs
29	RR262	1356	HA-Armi	PIWI	HA-Armi+reporter	51183511	37078836	261	OSCs
30	RR283	1356	HA-Armi	PIWI	HA-Armi+reporter	29499430	21966788	159	OSCs
31	RR288	1356	HA-Armi	PIWI	HA-Armi+reporter	43286861	34280499	305	OSCs
32	RR316	1356	HA-Armi	PIWI	HA-Armi+reporter	18711535	13177619	120	OSCs
33	RR326	1356	HA-Armi	PIWI	HA-Armi+reporter	28570361	20498247	255	OSCs
34	RR263	1356	NHA-Piwi	PIWI	NHA-Piwi+reporter	51643724	39016540	1377	OSCs
35	RR273	1356	NHA-Piwi	PIWI	NHA-Piwi+reporter	44093764	29917892	342	OSCs
36	RR289	1356	NHA-Piwi	PIWI	NHA-Piwi+reporter	42616883	33588717	562	OSCs
37	RR264	1356	HA-Piwi	PIWI	HA-Piwi+reporter	53534432	34098072	187	OSCs
38	RR274	1356	HA-Piwi	PIWI	HA-Piwi+reporter	44851033	29739316	130	OSCs
39	RR290	1356	HA-Piwi	PIWI	HA-Piwi+reporter	39945158	31223423	295	OSCs
40	RR265	1356	NHA-Yb	PIWI	NHA-Yb+reporter	53579640	40067532	17309	OSCs
41	RR275	1356	NHA-Yb	PIWI	NHA-Yb+reporter	54390352	36125467	2831	OSCs
42	RR291	1356	NHA-Yb	PIWI	NHA-Yb+reporter	42028244	32103252	6423	OSCs
43	RR266	1356	NHA-Yb_D-A	PIWI	NHA-Yb_D-A+reporter	54911076	40290173	10918	OSCs
44	RR276	1356	NHA-Yb_D-A	PIWI	NHA-Yb_D-A+reporter	57594057	36845719	3083	OSCs
45	RR292	1356	NHA-Yb_D-A	PIWI	NHA-Yb_D-A+reporter	35948297	26699976	3854	OSCs
46	RR267	1356	HA-Yb	PIWI	HA-Yb+reporter	52014710	37116720	333	OSCs
47	RR277	1356	HA-Yb	PIWI	HA-Yb+reporter	52417239	33983102	241	OSCs
48	RR293	1356	HA-Yb	PIWI	HA-Yb+reporter	35712923	26694116	314	OSCs
49	RR281	1356	NHA-Shu	PIWI	NHA-Shu+reporter	41971706	31046156	260	OSCs
50	RR317	1356	NHA-Shu	PIWI	NHA-Shu+reporter	19306659	13651642	217	OSCs
51	RR327	1356	NHA-Shu	PIWI	NHA-Shu+reporter	29532751	20063378	482	OSCs
52	RR282	1356	HA-Shu	PIWI	HA-Shu+reporter	43173520	31351982	168	OSCs
53	RR318	1356	HA-Shu	PIWI	HA-Shu+reporter	16312244	10814972	145	OSCs
54	RR328	1356	HA-Shu	PIWI	HA-Shu+reporter	29734879	20962371	507	OSCs
55	RR268	1356	NHA-LacZ	PIWI	NHA-LacZ+reporter	58693901	41895222	192	OSCs
56	RR278	1356	NHA-LacZ	PIWI	NHA-LacZ+reporter	53886947	36402286	209	OSCs
57	RR284	1356	NHA-LacZ	PIWI	NHA-LacZ+reporter	41346338	29271722	131	OSCs
58	RR294	1356	NHA-LacZ	PIWI	NHA-LacZ+reporter	30371094	23101548	130	OSCs
59	RR319	1356	NHA-LacZ	PIWI	NHA-LacZ+reporter	15991037	11473958	87	OSCs
60	RR329	1356	NHA-LacZ	PIWI	NHA-LacZ+reporter	29540065	20991740	513	OSCs
61	RR330	Uint	NHA-Armi	PIWI	NHA-Armi+Uint_reporter	34590390	27003447	4103	OSCs
62	RR336	Uint	NHA-Armi	PIWI	NHA-Armi+Uint_reporter	30164647	23330580	4098	OSCs
63	RR342	Uint	NHA-Armi	PIWI	NHA-Armi+Uint_reporter	29891764	23244606	5485	OSCs
64	RR332	Uint	NHA-LacZ	PIWI	NHA-LacZ+Uint_reporter	33557033	30506983	99	OSCs
65	RR338	Uint	NHA-LacZ	PIWI	NHA-LacZ+Uint_reporter	27527439	21591247	56	OSCs
66	RR344	Uint	NHA-LacZ	PIWI	NHA-LacZ+Uint_reporter	38698505	27981605	113	OSCs
67	RR331	Uint	HA-Armi	PIWI	HA-Armi+Uint_reporter	39084540	30663303	94	OSCs
68	RR337	Uint	HA-Armi	PIWI	HA-Armi+Uint_reporter	29479709	23203668	65	OSCs
69	RR343	Uint	HA-Armi	PIWI	HA-Armi+Uint_reporter	37766956	26375460	129	OSCs
70	RR333	Uless	NHA-Armi	PIWI	NHA-Armi+Uless_reporter	35598398	27502645	5337	OSCs
71	RR339	Uless	NHA-Armi	PIWI	NHA-Armi+Uless_reporter	27235027	21622592	5993	OSCs
72	RR345	Uless	NHA-Armi	PIWI	NHA-Armi+Uless_reporter	38550295	26897936	5155	OSCs
73	RR335	Uless	NHA-LacZ	PIWI	NHA-LacZ+Uless_reporter	30309272	24631386	100	OSCs
74	RR341	Uless	NHA-LacZ	PIWI	NHA-LacZ+Uless_reporter	22275030	16638821	60	OSCs
75	RR347	Uless	NHA-LacZ	PIWI	NHA-LacZ+Uless_reporter	36526722	25096181	81	OSCs
76	RR334	Uless	HA-Armi	PIWI	HA-Armi+Uless_reporter	33035863	26746046	122	OSCs
77	RR340	Uless	HA-Armi	PIWI	HA-Armi+Uless_reporter	24171785	21315419	311	OSCs
78	RR346	Uless	HA-Armi	PIWI	HA-Armi+Uless_reporter	38578390	25700468	101	OSCs
79	RR447	1356	Tj_NHA-Armi-wt	PIWI	Tj_NHA-Armi-wt+reporter	84257836	79001629	14339	fly follicle cells
80	RR454	1356	Tj_NHA-Armi-wt	PIWI	Tj_NHA-Armi-wt+reporter	43859140	42140185	8668	fly follicle cells
81	RR448	1356	Tj_NHA-Armi-DQ	PIWI	Tj_NHA-Armi-DQ+reporter	71691234	66806477	5484	fly follicle cells
82	RR455	1356	Tj_NHA-Armi-DQ	PIWI	Tj_NHA-Armi-DQ+reporter	45151002	41863801	2687	fly follicle cells
83	RR449	1356	Tj_NHA-Armi-GNT	PIWI	Tj_NHA-Armi-GNT+reporter	58525708	55610736	2328	fly follicle cells
84	RR456	1356	Tj_NHA-Armi-GNT	PIWI	Tj_NHA-Armi-GNT+reporter	58455675	55327818	2631	fly follicle cells
85	RR450	1356	Tj_HA-Armi-wt	PIWI	Tj_HA-Armi-wt+reporter	51700617	48118450	848	fly follicle cells
86	RR457	1356	Tj_HA-Armi-wt	PIWI	Tj_HA-Armi-wt+reporter	53507862	49591983	875	fly follicle cells
87	RR451	1356	Tj_NHA-Yb-wt	PIWI	Tj_NHA-Yb-wt+reporter	46788042	43472364	8715	fly follicle cells
88	RR458	1356	Tj_NHA-Yb-wt	PIWI	Tj_NHA-Yb-wt+reporter	54849211	50098874	10620	fly follicle cells
89	RR452	1356	Tj_NHA-Shu-wt	PIWI	Tj_NHA-Shu-wt+reporter	48641916	45984163	769	fly follicle cells
90	RR459	1356	Tj_NHA-Shu-wt	PIWI	Tj_NHA-Shu-wt+reporter	53986597	50064390	891	fly follicle cells
91	RR464	1356	NGT_HA-Armi-wt	Aub	NGT_HA-Armi-wt+reporter	47387134	43999969	144	fly germ cells
92	RR478	1356	NGT_HA-Armi-wt	Ago3	NGT_HA-Armi-wt+reporter	49288055	47333837	102	fly germ cells
93	RR471	1356	NGT_HA-Armi-wt	PIWI	NGT_HA-Armi-wt+reporter	49207459	47183695	405	fly germ cells
94	RR462	1356	NGT_NHA-Armi-DQ	Aub	NGT_NHA-Armi-DQ+reporter	57925546	55005522	525	fly germ cells
95	RR476	1356	NGT_NHA-Armi-DQ	Ago3	NGT_NHA-Armi-DQ+reporter	60041081	57287657	241	fly germ cells
96	RR469	1356	NGT_NHA-Armi-DQ	PIWI	NGT_NHA-Armi-DQ+reporter	58427439	55230679	1407	fly germ cells
97	RR463	1356	NGT_NHA-Armi-GNT	Aub	NGT_NHA-Armi-GNT+reporter	50241639	47339487	160	fly germ cells
98	RR477	1356	NGT_NHA-Armi-GNT	Ago3	NGT_NHA-Armi-GNT+reporter	48051625	45207328	110	fly germ cells
99	RR470	1356	NGT_NHA-Armi-GNT	PIWI	NGT_NHA-Armi-GNT+reporter	54839240	51901317	311	fly germ cells
100	RR461	1356	NGT_NHA-Armi-wt	Aub	NGT_NHA-Armi-wt+reporter	48648491	44407245	352	fly germ cells
101	RR475	1356	NGT_NHA-Armi-wt	Ago3	NGT_NHA-Armi-wt+reporter	44187186	41623558	128	fly germ cells
102	RR468	1356	NGT_NHA-Armi-wt	PIWI	NGT_NHA-Armi-wt+reporter	52578477	49875043	1054	fly germ cells
103	RR467	1356	NGT_NHA-Shu-wt	Aub	NGT_NHA-Shu-wt+reporter	53679402	50896046	394	fly germ cells
104	RR481	1356	NGT_NHA-Shu-wt	Ago3	NGT_NHA-Shu-wt+reporter	54426086	51785950	360	fly germ cells
105	RR474	1356	NGT_NHA-Shu-wt	PIWI	NGT_NHA-Shu-wt+reporter	53365614	50911597	642	fly germ cells
106	RR465	1356	NGT_NHA-Vret-wt	Aub	NGT_NHA-Vret-wt+reporter	49394959	46613180	71	fly germ cells
107	RR479	1356	NGT_NHA-Vret-wt	Ago3	NGT_NHA-Vret-wt+reporter	50916916	49021631	31	fly germ cells
108	RR472	1356	NGT_NHA-Vret-wt	PIWI	NGT_NHA-Vret-wt+reporter	58998144	55460665	114	fly germ cells
109	RR466	1356	NGT_NHA-Yb-wt	Aub	NGT_NHA-Yb-wt+reporter	50258500	47941737	79	fly germ cells
110	RR480	1356	NGT_NHA-Yb-wt	Ago3	NGT_NHA-Yb-wt+reporter	54395925	51518687	30	fly germ cells
111	RR473	1356	NGT_NHA-Yb-wt	PIWI	NGT_NHA-Yb-wt+reporter	47742276	44476638	257	fly germ cells

Reporter constructs used:

1347	Luc-LacZ
1355	Luc-Flam718-LacZ
1357	Luc-piRNA1-LacZ
1358	Luc-piRNA2-LacZ
1356	Luc-5BoxB-LacZ
Uless	Luc-5BoxB-LacZ with U-depleted regions
Uint	Luc-5BoxB-LacZ with Us at specific places
