# Supplementary material for: Recruitment of Armitage and Yb to a transcript triggers its phased processing into primary piRNAs in Drosophila ovaries
Source: PLoS Genet. 2017 Aug 21;13(8):e1006956. doi: 10.1371/journal.pgen.1006956 (PMC5578672; doi:10.1371/journal.pgen.1006956)
Supplement: S1 Protocols — A detailed description of protocols, materials and reporter sequences used in this study is given. (DOCX) [file pgen.1006956.s007.docx]

**S1 Protocols**

Detailed protocols, sequences of RNA oligos, information on fly lines used are given in this section.

**Antibodies used in this study**

Antibodies to all three *Drosophila* PIWI proteins used in this study were previously described [[1](#_ENREF_1)]. These include rabbit polyclonal antibodies (two rabbits: GJKO and GJLD) to *Drosophila* Piwi that were generated (EMBL Protein expression and purification core facility) against an insoluble antigen (Piwi antigen: 42-178 aa) produced in *E.coli* as an inclusion body. Single rabbits were used to generate the antibodies to *Drosophila* Aub and Ago3 (Aub antigen: 1-200 aa; Ago3 antigen:1-200 aa). Immunized rabbit sera were directly used for immunoprecipitation.

Other antibodies used are: anti-Armi (gift of M. Siomi) [[2](#_ENREF_2)], anti-Yb (gift of J. Brennecke) [[3](#_ENREF_3)], anti-Tubulin/actin (Abcam: ab6046), anti-His (Amersham: 27-4710-01) and anti-MBP (NEB: E8032S) antibodies. HA tagged proteins were detected by mouse anti-HA (42F3; gift of M. Buehler) and rabbit polyclonal anti-HA (Santa Cruz: sc-805, HA-probe Y-11).

For immunofluorescence studies the following secondary antibodies were used: anti-rabbit (Life Technologies; Alexa Fluor 488, A11034), anti-rabbit (Life Technologies; Alexa Fluor 594, A11037) and anti-mouse (Life Technologies; Alexa Fluor 594, A11005). The following secondary antibodies conjugated to Horse Radish Peroxidase were used for Western analyses: anti-rabbit IgG HRP-linked antibody (GE Healthcare; NA934), anti-mouse IgG HRP-linked (GE Healthcare; NA931).

**Constructs for OSC experiments**

For expression in the *Drosophila* ovarian somatic cell (OSC) cultures [[4](#_ENREF_4)], we used the pAC5.1 vector (Life Technologies) driving expression from the fly *actin* promoter [[1](#_ENREF_1)]. For expression of either HA-tag (pAC-HA) or N-HA-tag fusions (pAC-NHA), the pAC5.1 vector was further modified to add the necessary coding sequences. The HA tag is for detection of the expressed protein and the λN-peptide is for artificially tethering the fusion protein to a transcript containing BoxB sequences [[5](#_ENREF_5)].

The following sequences were chemically synthesized (ShineGene Bio-Technologies, Inc., Shanghai, China) and cloned as the BoxB reporter transcripts in the KpnI and XbaI restrictions sites of the pAC5.1 vector. The Original Reporter-5BoxB contained a combination of sequences derived from the firefly luciferase from *Photinus pyralis* and LacZ, with five BoxB hairpins placed in between at HindIII-NotI site. These sequences were also used for mapping the reads from deep sequencing libraries.

Original Reporter-5BoxB

**GGTACC**GGCATTCCGGTACTGTTGGTAAAGCCACCATGGAAGACGCCAAAAACATAAAGAAAGGCCCGGCGCCATTCTATCCGCTGGAAGATGGAACCGCTGGAGAGCAACTGCATAAGGCTATGAAGAGATACGCCCTGGTTCCTGGAACAATTGCTTTTACAGATGCACATATCGAGGTGGACATCACTTACGCTGAGTACTTCGAAATGTCCGTTCGGTTGGCAGAAGCTATGAAACGATATGGGCTGAATACAAATCACAGAATCGTCGTATGCAGTGAAAACTCTCTTCAATTCTTTATGCCGGTGTTGGGCGCGTTATTTATCGGAGTTGCAGTTGCGCCCGCGAACGACATTTATAATGAACGTGAATTGCTCAACAGTATGGGCATTTCGCAGCCTACCGTGGTGTTCGTTTCCAAAAAGGGGTTGCAAAAAATTTTGAACGTGCAAAAAAAGCTCCCAATCATCCAAAAAATTATTATCATGGATTCTAAAACGGATTACCAGGGATTTCAGTCGATGTACACGTTCGTCACATCTCATCTACCTCCCGGTTTTAATGAATACGATTTTGTGCCAGAAGCTTTAAGTCCAACTACTAAACTGGGGATTCCTGGGCCCTGAAGAAGGGCCCCTCGACTAAGTCCAACTACTAAACTGGGCCCTGAAGAAGGGCCCATATAGGGCCCTGAAGAAGGGCCCTATCGAGGATATTATCTCGACTAAGTCCAACTACTAAACTGGGCCCTGAAGAAGGGCCCATATAGGGCCCTGAAGAAGGGCCCTATCGAGGATATTATCTCGAGGCGGCCGCGTCGTTTTACAACGTCGTGACTGGGAAAACCCTGGCGTTACCCAACTTAATCGCCTTGCAGCACATCCCCCTTTCGCCGGCTGGCGTAATAGCGAAGAGGCCCGCACCGATCGCCCTTCCCAACAGTTGCGCAGCCTGAAAGGCGAAAGGCGCTTTGCCTGGTTTCCGGCACCAGAAGCGGTGCCGGAAAGCTGGCTGGAGTGCGATCTTCCTGAGGCCGATACTGTCGTCGTCCCCTCAAACTGGCAGAAGCACGGTTACGAAGCGCCCATCTACACCAACGTAACCTATCCCATTACGGTCAATCCGCCGTTTGTTCCCACGGAGAATCCGACGGGTTGTTACTCGCTCACATTTAAAGTTGAAGAAAGCTGGCTACAGGAAGGCCAGACGCGAATTATTTTTGAAGGCGTTAACTCGGCGTTTCATCTGTGGTGCAACGGGCGCTGGGTCGGTTACGGCCAGGACAGTCGTTTGCCGTCTGAATTTGACCTGAGCGCATTTTTACGCGCCGGAGAAAACCGCCTCGCGGTGAAGGTGCTGCGTTGGAGTGACGGCAGTTATCTGGAAGATCAGGATAAGTGGCGGAAGAGCGGCATTTTCCGTGACGTCTCGTTGCTGCATAAACCGACTACACAAATCAGCGATTTCCAAGTTGCCACTCGCTTTAAAGAAGATTTCAGCCGCGCTGTACTGG**C**TCGAGTCTAGAGGGCCCGCGGTTCGAAGGTAAGCCTATCCCTAACCCTCTCCTCGGTCTCGATTCTACGCGTACCGGTCATCATCACCATCACCATTGAGTTTAAACCCGCTGATCAGCCTCGACTGTGCCTTCTAAGGCCTGAGCTCGCTGATCAGCCTCGATCGAGGATCCAGACATGATAAGATACATTGATGAGTTTGGACAAACCACAACTAGAATGCAGTGAAAAAAATGCTTTATTTGTGAAATTTGTGATGCTATTGCTTTATTTGTAACCATTATAAGCTGCAATAAACAAGTTAACAACAACAATTGCATTCATTTTATGTTTCAGGTTCAGGGGGAGG

U-less 5BoxB reporter (same as Original Reporter-5BoxB, but having two patches of no-U sequences)

GGTACCGGCATTCCGGTACTGTTGGTAAAGCCACCATGGAAGACGCCAAAAACATAAAGAAAGGCCCGGCGCCATTCTATCCGCTGGAAGATGGAACCGCTGGAGAGCAACTGCATAAGGCTATGAAGAGATACGCCCTGGTTCCTGGAACAATTGCTTTTACAGATGCACATATCGAGGTGGACATCACTTACGCTGAGTACTTCGAAATGTCCGTTCGGTTGGCAGAAGCTATGAAACGATATGGGCTGAATACAAATCACAGAATCGTCGTATGCAGTGAAAACTCTCTTCAATTCTTTATGCCGGTGTTGGGCGCGTTATTTATCGGAGTTGCAGTTGCGCCCGCGAACGACATTTATAATGAACGTGAATTGCTCAACAGTATGGGCATTTCGCAGCCTACCGTGGTGTTCGTTTCCAAAAAGGGGTTGCAAAAAATTTTGAACGTGCAAAAAAAGCTCCCAATCATCCAAAAAATTATTATCATGGATTCTAAAACGGATTACCAGGGATTTCAGTCGATGTACACGTTCGTCACATCTCATCTACCTCCCGGTTTTAATGAATACGATTTTGTGCCAGAAGCTTTAAGTCCAACTACTAAACTGGGGATTCCTGGGCCCTGAAGAAGGGCCCCTCGACTAAGTCCAACTACTAAACTGGGCCCTGAAGAAGGGCCCATATAGGGCCCTGAAGAAGGGCCCTATCGAGGATATTATCTCGACTAAGTCCAACTACTAAACTGGGCCCTGAAGAAGGGCCCATATAGGGCCCTGAAGAAGGGCCCTATCGAGGATATTATCTCGAGGCGGCCGCGTCGTTTTACAACGTCGTGACTGGGAAAACCCTGGCGTTACCCAACTTAATCGCCTTGCAGCACATCCCCCTTTCGCCGGCTGGCGTAATAGCGAAGAGGCCCGCACCGAACGCCCAGCCCAACAGAAGCGCAGCCAGAAAGGCGAAAGGCGCAACGCCAGGTTTCCGGCACCAGAAGCGGTGCCGGAAAGCTGGCTGGAGTGCGATCTTCCTGAGGCCGATACTGTCGTCGACCCCGCAAACAGGCAGAAGCACGGCGACGAAGCGCCCAGCGACACCAACGGAACCGAGCCCAGCACGGACAAGCCGCCGAACGAGCCCACGGAGAAGCCGACGGGACGAGACACGCACACAGAGAAAGCCGAAGAAAGCAGGCAACAGGAAGGCCAGACGCGAACGACAGCAGAAGGCGACAACACGGCGAGACAGCAGCGGAGCAACGGGCGCAGGGACGGACACGGCCAGGACAGTCGTTTGCCGTCTGAATTTGACCTGAGCGCATTTTTACGCGCCGGAGAAAACCGCCTCGCGGTGAAGGTGCTGCGTTGGAGTGACGGCAGTTATCTGGAAGATCAGGATAAGTGGCGGAAGAGCGGCATTTTCCGTGACGTCTCGTTGCTGCATAAACCGACTACACAAATCAGCGATTTCCAAGTTGCCACTCGCTTTAAAGAAGATTTCAGCCGCGCTGTACTGGCTCGAGTCTAGAGGGCCCGCGGTTCGAAGGTAAGCCTATCCCTAACCCTCTCCTCGGTCTCGATTCTACGCGTACCGGTCATCATCACCATCACCATTGAGTTTAAACCCGCTGATCAGCCTCGACTGTGCCTTCTAAGGCCTGAGCTCGCTGATCAGCCTCGATCGAGGATCCAGACATGATAAGATACATTGATGAGTTTGGACAAACCACAACTAGAATGCAGTGAAAAAAATGCTTTATTTGTGAAATTTGTGATGCTATTGCTTTATTTGTAACCATTATAAGCTGCAATAAACAAGTTAACAACAACAATTGCATTCATTTTATGTTTCAGGTTCAGGGGGAGG

U-interval 5BoxB reporter (same as Original Reporter-5BoxB, but having Us spaced at varying intervals)

GGTACCGGCATTCCGGTACTGTTGGTAAAGCCACCATGGAAGACGCCAAAAACATAAAGAAAGGCCCGGCGCCATTCTATCCGCTGGAAGATGGAACCGCTGGAGAGCAACTGCATAAGGCTATGAAGAGATACGCCCTGGTTCCTGGAACAATTGCTTTTACAGATGCACATATCGAGGTGGACATCACTTACGCTGAGTACTTCGAAATGTCCGTTCGGTTGGCAGAAGCTATGAAACGATATGGGCTGAATACAAATCACAGAATCGTCGTATGCAGTGAAAACTCTCTTCAATTCTTTATGCCGGTGTTGGGCGCGTTATTTATCGGAGTTGCAGTTGCGCCCGCGAACGACATTTATAATGAACGTGAATTGCTCAACAGTATGGGCATTTCGCAGCCTACCGTGGTGTTCGTTTCCAAAAAGGGGTTGCAAAAAATTTTGAACGTGCAAAAAAAGCTCCCAATCATCCAAAAAATTATTATCATGGATTCTAAAACGGATTACCAGGGATTTCAGTCGATGTACACGTTCGTCACATCTCATCTACCTCCCGGTTTTAATGAATACGATTTTGTGCCAGAAGCTTTAAGTCCAACTACTAAACTGGGGATTCCTGGGCCCTGAAGAAGGGCCCCTCGACTAAGTCCAACTACTAAACTGGGCCCTGAAGAAGGGCCCATATAGGGCCCTGAAGAAGGGCCCTATCGAGGATATTATCTCGACTAAGTCCAACTACTAAACTGGGCCCTGAAGAAGGGCCCATATAGGGCCCTGAAGAAGGGCCCTATCGAGGATATTATCTCGAGGCGGCCGCGACGAGATACAACGGCGCGACCGGGAATACCCAGGCGGCACCCAACAAAACTGCCCCGCAGCACACCCCCCCCACGCCGTCGGGCGAAAGAGCGAAGAGGCCCGCACCGACTGCCCAGCCCAACAGAAGCGCTGCCAGAAAGGCGAAAGGCGCAACGTCAGGGGCCCGGCACCAGAAGCGGAGCCGTAAAGCCGGCCGGAGAGCGAGCCACCCGAGGCCTAGACGGGCGCCGACCCCGCAATCAGGCAGAAGCACGGCGACGAAGCGTCCAGCGACACCAACGGAACCGAGCCCAGCTCGGACAAGCCGCCGAACGAGCCCACGGAGAAGCTGACGGGACGAGACACGCACACATAGAAAGCCGAAGAAAGCAGGCAACAGTAAGGCCAGACGCGAACGACAGCAGAAGGCGTCAACACGGCGAGACAGCAGCGGAGCAACGGGCGCTTTGACGGACACGGCCAGGACAGCCGTTTGCCGCCGGAAAGGGACCAGAGCGCATTTCAACGCGCCGGAGAAAACCGCCCCGCGGCTAAGGGGCGGCGGCGGAGAGACGGCAGGAAGCGGGAAGAGTAGGAGAAGCGGCGGAAGAGCGGCACAAGCCGCGACGGCACGGAGTCGCACAAACCGACGACACAAACCAGCGAACACCAAGCAGCCACACGCCATAAAGAAGAGAACAGCCGCGCCGAACTGGCCCGAGTCTAGAGGGCCCGCGGTTCGAAGGTAAGCCTATCCCTAACCCTCTCCTCGGTCTCGATTCTACGCGTACCGGTCATCATCACCATCACCATTGAGTTTAAACCCGCTGATCAGCCTCGACTGTGCCTTCTAAGGCCTGAGCTCGCTGATCAGCCTCGATCGAGGATCCAGACATGATAAGATACATTGATGAGTTTGGACAAACCACAACTAGAATGCAGTGAAAAAAATGCTTTATTTGTGAAATTTGTGATGCTATTGCTTTATTTGTAACCATTATAAGCTGCAATAAACAAGTTAACAACAACAATTGCATTCATTTTATGTTTCAGGTTCAGGGGGAGG

Reporter **lacking** BoxB (same as Original Reporter-5BoxB, but lacking BoxBs)

**GGTACC**GGCATTCCGGTACTGTTGGTAAAGCCACCATGGAAGACGCCAAAAACATAAAGAAAGGCCCGGCGCCATTCTATCCGCTGGAAGATGGAACCGCTGGAGAGCAACTGCATAAGGCTATGAAGAGATACGCCCTGGTTCCTGGAACAATTGCTTTTACAGATGCACATATCGAGGTGGACATCACTTACGCTGAGTACTTCGAAATGTCCGTTCGGTTGGCAGAAGCTATGAAACGATATGGGCTGAATACAAATCACAGAATCGTCGTATGCAGTGAAAACTCTCTTCAATTCTTTATGCCGGTGTTGGGCGCGTTATTTATCGGAGTTGCAGTTGCGCCCGCGAACGACATTTATAATGAACGTGAATTGCTCAACAGTATGGGCATTTCGCAGCCTACCGTGGTGTTCGTTTCCAAAAAGGGGTTGCAAAAAATTTTGAACGTGCAAAAAAAGCTCCCAATCATCCAAAAAATTATTATCATGGATTCTAAAACGGATTACCAGGGATTTCAGTCGATGTACACGTTCGTCACATCTCATCTACCTCCCGGTTTTAATGAATACGATTTTGTGCCAGGCGGCCGCGTCGTTTTACAACGTCGTGACTGGGAAAACCCTGGCGTTACCCAACTTAATCGCCTTGCAGCACATCCCCCTTTCGCCGGCTGGCGTAATAGCGAAGAGGCCCGCACCGATCGCCCTTCCCAACAGTTGCGCAGCCTGAAAGGCGAAAGGCGCTTTGCCTGGTTTCCGGCACCAGAAGCGGTGCCGGAAAGCTGGCTGGAGTGCGATCTTCCTGAGGCCGATACTGTCGTCGTCCCCTCAAACTGGCAGAAGCACGGTTACGAAGCGCCCATCTACACCAACGTAACCTATCCCATTACGGTCAATCCGCCGTTTGTTCCCACGGAGAATCCGACGGGTTGTTACTCGCTCACATTTAAAGTTGAAGAAAGCTGGCTACAGGAAGGCCAGACGCGAATTATTTTTGAAGGCGTTAACTCGGCGTTTCATCTGTGGTGCAACGGGCGCTGGGTCGGTTACGGCCAGGACAGTCGTTTGCCGTCTGAATTTGACCTGAGCGCATTTTTACGCGCCGGAGAAAACCGCCTCGCGGTGAAGGTGCTGCGTTGGAGTGACGGCAGTTATCTGGAAGATCAGGATAAGTGGCGGAAGAGCGGCATTTTCCGTGACGTCTCGTTGCTGCATAAACCGACTACACAAATCAGCGATTTCCAAGTTGCCACTCGCTTTAAAGAAGATTTCAGCCGCGCTGTACTGG**C**TCGAGTCTAGAGGGCCCGCGGTTCGAAGGTAAGCCTATCCCTAACCCTCTCCTCGGTCTCGATTCTACGCGTACCGGTCATCATCACCATCACCATTGAGTTTAAACCCGCTGATCAGCCTCGACTGTGCCTTCTAAGGCCTGAGCTCGCTGATCAGCCTCGATCGAGGATCCAGACATGATAAGATACATTGATGAGTTTGGACAAACCACAACTAGAATGCAGTGAAAAAAATGCTTTATTTGTGAAATTTGTGATGCTATTGCTTTATTTGTAACCATTATAAGCTGCAATAAACAAGTTAACAACAACAATTGCATTCATTTTATGTTTCAGGTTCAGGGGGAGG

Reporter with PTS instead of 5BoxB (Luc-*flam*718-LacZ)

The reporter has a 1-718 nt fragment of the *flamenco* piRNA cluster precursor transcript instead of 5BoxB hairpins in between the luciferase and LacZ regions.

GGTACCGGCATTCCGGTACTGTTGGTAAAGCCACCATGGAAGACGCCAAAAACATAAAGAAAGGCCCGGCGCCATTCTATCCGCTGGAAGATGGAACCGCTGGAGAGCAACTGCATAAGGCTATGAAGAGATACGCCCTGGTTCCTGGAACAATTGCTTTTACAGATGCACATATCGAGGTGGACATCACTTACGCTGAGTACTTCGAAATGTCCGTTCGGTTGGCAGAAGCTATGAAACGATATGGGCTGAATACAAATCACAGAATCGTCGTATGCAGTGAAAACTCTCTTCAATTCTTTATGCCGGTGTTGGGCGCGTTATTTATCGGAGTTGCAGTTGCGCCCGCGAACGACATTTATAATGAACGTGAATTGCTCAACAGTATGGGCATTTCGCAGCCTACCGTGGTGTTCGTTTCCAAAAAGGGGTTGCAAAAAATTTTGAACGTGCAAAAAAAGCTCCCAATCATCCAAAAAATTATTATCATGGATTCTAAAACGGATTACCAGGGATTTCAGTCGATGTACACGTTCGTCACATCTCATCTACCTCCCGGTTTTAATGAATACGATTTTGTGCCAGAAGCTTAGTTGCTTTATGACGCCGGGCGCGTGCGCATTTAAAACAATTCTCGAAAGAAATGTTCACAAATTAGTTTAAATTCGAAAACGTTTCGGTATGGACGCAAGAAAAAGGAATTCTAAGAAATAGAAATTCTAAGCGAAACAACAACGAAAAAGGAAAATAATTTAAATAGGGATAATAATATACAAAAACATAACAACAAAAACACAACGGTATTAAGGAATCTGTTTCAGAATTTCAGTGAGTTAAATCTGTTAAAAAGTTCTGCGGTTAATTTGTTTGTTTCTTTAACAAAAAACGTAATCTAATATTTTAATTATTATCATCAGCTCAGCAGCAGTGTAGAATATATTGGCCTAAAATACTAGATATTTTATGTTAATATATTAAATTCGAGTGACTTACGTCACCCAAGATTTTGTACATTTTCAAGCGGTAAGTGTTAAGTCAGCGCGTTCGTATTTATGGGACATTGGAGATTTGTACATGGATGCTAGTGGGGGAACGGCTATTAAAATTTCGCTGACTGGTTGGGTGTCAAGTTCGCAAGACTGAAGTTTCGATGACTAAGGGTATCCTCCTACTGTTTTGGAATTGTTTATGCTGGATTGTATTTTATGTTAATGTGTGGAATTTGGTTGGGTCACTTTTCTCGATGTGGTTCGATTGAAGTTTTCAACCTAGTTTTAGTAATTGTAGTTTCATATAGAATAGGCAGGCTGCGGCCGCGTCGTTTTACAACGTCGTGACTGGGAAAACCCTGGCGTTACCCAACTTAATCGCCTTGCAGCACATCCCCCTTTCGCCGGCTGGCGTAATAGCGAAGAGGCCCGCACCGATCGCCCTTCCCAACAGTTGCGCAGCCTGAAAGGCGAAAGGCGCTTTGCCTGGTTTCCGGCACCAGAAGCGGTGCCGGAAAGCTGGCTGGAGTGCGATCTTCCTGAGGCCGATACTGTCGTCGTCCCCTCAAACTGGCAGAAGCACGGTTACGAAGCGCCCATCTACACCAACGTAACCTATCCCATTACGGTCAATCCGCCGTTTGTTCCCACGGAGAATCCGACGGGTTGTTACTCGCTCACATTTAAAGTTGAAGAAAGCTGGCTACAGGAAGGCCAGACGCGAATTATTTTTGAAGGCGTTAACTCGGCGTTTCATCTGTGGTGCAACGGGCGCTGGGTCGGTTACGGCCAGGACAGTCGTTTGCCGTCTGAATTTGACCTGAGCGCATTTTTACGCGCCGGAGAAAACCGCCTCGCGGTGAAGGTGCTGCGTTGGAGTGACGGCAGTTATCTGGAAGATCAGGATAAGTGGCGGAAGAGCGGCATTTTCCGTGACGTCTCGTTGCTGCATAAACCGACTACACAAATCAGCGATTTCCAAGTTGCCACTCGCTTTAAAGAAGATTTCAGCCGCGCTGTACTGGCTCGAGTCTAGAGGGCCCGCGGTTCGAAGGTAAGCCTATCCCTAACCCTCTCCTCGGTCTCGATTCTACGCGTACCGGTCATCATCACCATCACCATTGAGTTTAAACCCGCTGATCAGCCTCGACTGTGCCTTCTAAGGCCTGAGCTCGCTGATCAGCCTCGATCGAGGATCCAGACATGATAAGATACATTGATGAGTTTGGACAAACCACAACTAGAATGCAGTGAAAAAAATGCTTTATTTGTGAAATTTGTGATGCTATTGCTTTATTTGTAACCATTATAAGCTGCAATAAACAAGTTAACAACAACAATTGCATTCATTTTATGTTTCAGGTTCAGGGGGAGG

Reporter with piRNA binding site

We also created reporters with a perfectly complementary binding site for abundant Piwi-bound piRNAs. Briefly, we identified two most abundant piRNA sequences frequently found in multiple Piwi-bound piRNA libraries prepared from *Drosophila* OSC cultures. We inserted a single perfectly complementary binding site for a piRNA in between the firefly luciferase and LacZ sequences. The required reporter sequence was chemically synthesized (ShineGene Bio-Technologies, Inc., Shanghai, China).

Reporter with piRNA-1 target site

GGTACCGGCATTCCGGTACTGTTGGTAAAGCCACCATGGAAGACGCCAAAAACATAAAGAAAGGCCCGGCGCCATTCTATCCGCTGGAAGATGGAACCGCTGGAGAGCAACTGCATAAGGCTATGAAGAGATACGCCCTGGTTCCTGGAACAATTGCTTTTACAGATGCACATATCGAGGTGGACATCACTTACGCTGAGTACTTCGAAATGTCCGTTCGGTTGGCAGAAGCTATGAAACGATATGGGCTGAATACAAATCACAGAATCGTCGTATGCAGTGAAAACTCTCTTCAATTCTTTATGCCGGTGTTGGGCGCGTTATTTATCGGAGTTGCAGTTGCGCCCGCGAACGACATTTATAATGAACGTGAATTGCTCAACAGTATGGGCATTTCGCAGCCTACCGTGGTGTTCGTTTCCAAAAAGGGGTTGCAAAAAATTTTGAACGTGCAAAAAAAGCTCCCAATCATCCAAAAAATTATTATCATGGATTCTAAAACGGATTACCAGGGATTTCAGTCGATGTACACGTTCGTCACATCTCATCTACCTCCCGGTTTTAATGAATACGATTTTGTGCCAGAAGCTTGGGAGCGACGATGGCAGAGTCAGCAACGGAGCGGCCGCGTCGTTTTACAACGTCGTGACTGGGAAAACCCTGGCGTTACCCAACTTAATCGCCTTGCAGCACATCCCCCTTTCGCCGGCTGGCGTAATAGCGAAGAGGCCCGCACCGATCGCCCTTCCCAACAGTTGCGCAGCCTGAAAGGCGAAAGGCGCTTTGCCTGGTTTCCGGCACCAGAAGCGGTGCCGGAAAGCTGGCTGGAGTGCGATCTTCCTGAGGCCGATACTGTCGTCGTCCCCTCAAACTGGCAGAAGCACGGTTACGAAGCGCCCATCTACACCAACGTAACCTATCCCATTACGGTCAATCCGCCGTTTGTTCCCACGGAGAATCCGACGGGTTGTTACTCGCTCACATTTAAAGTTGAAGAAAGCTGGCTACAGGAAGGCCAGACGCGAATTATTTTTGAAGGCGTTAACTCGGCGTTTCATCTGTGGTGCAACGGGCGCTGGGTCGGTTACGGCCAGGACAGTCGTTTGCCGTCTGAATTTGACCTGAGCGCATTTTTACGCGCCGGAGAAAACCGCCTCGCGGTGAAGGTGCTGCGTTGGAGTGACGGCAGTTATCTGGAAGATCAGGATAAGTGGCGGAAGAGCGGCATTTTCCGTGACGTCTCGTTGCTGCATAAACCGACTACACAAATCAGCGATTTCCAAGTTGCCACTCGCTTTAAAGAAGATTTCAGCCGCGCTGTACTGGCTCGAGTCTAGAGGGCCCGCGGTTCGAAGGTAAGCCTATCCCTAACCCTCTCCTCGGTCTCGATTCTACGCGTACCGGTCATCATCACCATCACCATTGAGTTTAAACCCGCTGATCAGCCTCGACTGTGCCTTCTAAGGCCTGAGCTCGCTGATCAGCCTCGATCGAGGATCCAGACATGATAAGATACATTGATGAGTTTGGACAAACCACAACTAGAATGCAGTGAAAAAAATGCTTTATTTGTGAAATTTGTGATGCTATTGCTTTATTTGTAACCATTATAAGCTGCAATAAACAAGTTAACAACAACAATTGCATTCATTTTATGTTTCAGGTTCAGGGGGAGG

Reporter with piRNA-2 target site

GGTACCGGCATTCCGGTACTGTTGGTAAAGCCACCATGGAAGACGCCAAAAACATAAAGAAAGGCCCGGCGCCATTCTATCCGCTGGAAGATGGAACCGCTGGAGAGCAACTGCATAAGGCTATGAAGAGATACGCCCTGGTTCCTGGAACAATTGCTTTTACAGATGCACATATCGAGGTGGACATCACTTACGCTGAGTACTTCGAAATGTCCGTTCGGTTGGCAGAAGCTATGAAACGATATGGGCTGAATACAAATCACAGAATCGTCGTATGCAGTGAAAACTCTCTTCAATTCTTTATGCCGGTGTTGGGCGCGTTATTTATCGGAGTTGCAGTTGCGCCCGCGAACGACATTTATAATGAACGTGAATTGCTCAACAGTATGGGCATTTCGCAGCCTACCGTGGTGTTCGTTTCCAAAAAGGGGTTGCAAAAAATTTTGAACGTGCAAAAAAAGCTCCCAATCATCCAAAAAATTATTATCATGGATTCTAAAACGGATTACCAGGGATTTCAGTCGATGTACACGTTCGTCACATCTCATCTACCTCCCGGTTTTAATGAATACGATTTTGTGCCAGAAGCTTATGAAACAAAACGGAGAATGCTATAGTCGAGCGGCCGCGTCGTTTTACAACGTCGTGACTGGGAAAACCCTGGCGTTACCCAACTTAATCGCCTTGCAGCACATCCCCCTTTCGCCGGCTGGCGTAATAGCGAAGAGGCCCGCACCGATCGCCCTTCCCAACAGTTGCGCAGCCTGAAAGGCGAAAGGCGCTTTGCCTGGTTTCCGGCACCAGAAGCGGTGCCGGAAAGCTGGCTGGAGTGCGATCTTCCTGAGGCCGATACTGTCGTCGTCCCCTCAAACTGGCAGAAGCACGGTTACGAAGCGCCCATCTACACCAACGTAACCTATCCCATTACGGTCAATCCGCCGTTTGTTCCCACGGAGAATCCGACGGGTTGTTACTCGCTCACATTTAAAGTTGAAGAAAGCTGGCTACAGGAAGGCCAGACGCGAATTATTTTTGAAGGCGTTAACTCGGCGTTTCATCTGTGGTGCAACGGGCGCTGGGTCGGTTACGGCCAGGACAGTCGTTTGCCGTCTGAATTTGACCTGAGCGCATTTTTACGCGCCGGAGAAAACCGCCTCGCGGTGAAGGTGCTGCGTTGGAGTGACGGCAGTTATCTGGAAGATCAGGATAAGTGGCGGAAGAGCGGCATTTTCCGTGACGTCTCGTTGCTGCATAAACCGACTACACAAATCAGCGATTTCCAAGTTGCCACTCGCTTTAAAGAAGATTTCAGCCGCGCTGTACTGGCTCGAGTCTAGAGGGCCCGCGGTTCGAAGGTAAGCCTATCCCTAACCCTCTCCTCGGTCTCGATTCTACGCGTACCGGTCATCATCACCATCACCATTGAGTTTAAACCCGCTGATCAGCCTCGACTGTGCCTTCTAAGGCCTGAGCTCGCTGATCAGCCTCGATCGAGGATCCAGACATGATAAGATACATTGATGAGTTTGGACAAACCACAACTAGAATGCAGTGAAAAAAATGCTTTATTTGTGAAATTTGTGATGCTATTGC

**Tagged-fusion protein constructs for OSC**

For fusion proteins, the following coding sequences were cloned into pAC-HA or pAC-NHA plasmids.

*Drosophila* protein sequences were:

Armitage (Armi; 1-1188 aa).

Armi^DQAG^ mutant with E863Q mutation.

Armi^GNT^ mutant with K729N mutation.

Armi^Helicase^ (495-1151 aa) RNA helicase domain alone.

Armi^N-term^ (1-448 aa) N-terminal region alone

Yb (1-1042 aa)

Yb^D537A^ RNA binding domain mutant with D537A mutation [[6](#_ENREF_6)]

Shutdown (1-455 aa)

Piwi (1-843 aa)

Other sequence used was:

LacZ

**Constructs and crosses for transgenic *Drosophila* experiments**

For fly ovarian somatic and germline expressions, these constructs were prepared:

The pUASp-attB-delK10- vector (has *P* transposase promoter, the first intron and the *fs(1)K10* 3ʹ UTR; drives expression in ovarian germline) was used to clone the following sequences into the NheI and NotI restriction sites. This vector has the UAS *Gal4* binding site upstream of *P* transposase promoter, that respond to the GAL4 transcription factor. HA or NHA tag sequences were also added into KpnI and NheI restriction site at the 5ʹ end of protein coding sequence.

*Drosophila* protein sequences were cloned:

Armitage (Armi; 1-1188 aa) (both HA- and NHA-tagged)

Armi^DQAG^ mutant with E863Q mutation (NHA-tag only)

Armi^GNT^ mutant with K729N mutation (NHA-tag only)

Yb (1-1042 aa) (NHA-tag only)

Shutdown (1-455 aa) (NHA-tag only)

Other sequence used was:

Original Reporter-5BoxB (cloned into KpnI and XbaI restriction site).

Creation of transgenic flies

For creating transgenic fly lines, the coding sequences for NHA- or HA-tagged fusions of the proteins mentioned above were inserted into the pUASp_attB_delK10 plasmid containing the *white+* gene marker. These were used for site-specific integration (BestGene, Inc) in the *Drosophila* genome using the PhiC31 (ΦC31) integrase-mediated transgenesis system [[7](#_ENREF_7), [8](#_ENREF_8)]. The fly line used for integration is BDSC#9744 (*attP* landing site VK27 situated on the third chromosome at cytological location 89E11 carrying the *yellow+* gene marker, genotype: PBac(yellow[+]-attP-9A)VK00027 [[9](#_ENREF_9)].

Similarly, the reporter gene *Reporter-5BoxB* (see sequence details above in Original Reporter-5BoxB) was cloned into the pUASp_attB_delK10 plasmid and integrated (BestGene, Inc) in BDSC#9752 (attP landing site VK37 situated on the second chromosome at cytological location 22A3, genotype: PBac(yellow[+]-attP-3B)VK00037) or in BDSC#9736.

Ovarian soma expression

For fly ovarian soma expression, a recombinant line carrying the ovarian soma-specific driver *traffic jam*-Gal4 (*tj*-Gal4) (gift of A. Pelisson), and the UASp- *Reporter-5BoxB* was created by classical meiotic recombination in the y[1]w[1118] genetic background. Then homozygous females yw;*tj*-Gal4,UASp- *Reporter-5BoxB* were crossed with males from each line carrying the transgene expressing one of the various fusion proteins: UASp-NHA-Armi-WT, UASp-NHA-Armi^DQAG^, UASp-NHA-Armi^GNT^, UASp-HA-Armi-WT, UASp-NHA-Yb or UASp-NHA-Shu. The resulting F1 female progeny of the following genotype: yw;tj-Gal4,UASp- *Reporter-5BoxB*/+;UASp-Fusion protein/+ was selected and analyzed for piRNA expression or protein expression in the ovaries.

Ovarian germ line expression

For fly ovarian germ line expression, each transgene allowing for the expression of fusion proteins were individually combined with the ovarian germline driver line NGT-Gal4 (gift of J. Brennecke) [[3](#_ENREF_3)] by classical genetic crosses using balancers of the second (CyO) and third (TM6b) chromosomes from a multiple balanced line y[1],w[1118]; If/CyO; Sb/TM6b. Females yw;NGT-Gal4/(CyO); UASp-fusion protein/(TM6b) carrying or not CyO and TM6b balancers (to avoid dominant partial sterility induced by the expression of some of the transgenes) were then crossed with UASp- *Reporter-5BoxB* gene and the resulting F1 progeny carrying the three transgenes (yw;NGT-Gal4/UASp- *Reporter-5BoxB*;UASp-fusion protein/+) was selected and analyzed for piRNA expression or protein expression in the ovaries.

Alternatively, in a second set of experiments, a homozygous, fully viable and fertile recombinant line yw;NGTGal4,UASp- *Reporter-5BoxB* was created by meiotic recombination and crossed with each of the UASp-fusion protein lines. The resulting progeny of the following genotype yw;NGT-Gal4,UASp- *Reporter-5BoxB*/+; UASp-fusion protein/+ was analyzed for piRNA or protein expression in the ovaries with similar results.

**OSC Cell culture and electroporation**

*Drosophila* ovarian somatic cell (OSC; gift of Dr. M. Siomi, University of Tokyo) culture system is representative of the fly ovarian somatic follicle cells [[4](#_ENREF_4)]. OSCs were cultured in 75 cm flasks and grown to 80% confluence. Approximately 3.5x10^6^ cells were used for each electroporation reaction using Cell Line Nucleofector Kit V (Lonza, Cat No. VCA-1003) and were plated in 6-well plate.

Briefly, OSCs were grown in Shields and Sang M3 Insect Medium (Sigma, S8398) with supplements (10% heat inactivated FBS, Insulin 1 µl/ml (I9278 Sigma), GSH (G6013, Sigma, 140 µl/ml from a stock of 50 mg/ml), 1 ml Penicillin-Streptomycin/100 ml medium and 3-4 days old female fly extract). Once the cells were 80% confluence, they were harvested by mild trypsinization.

Cells were counted and ~3.5x10^6^ cells were taken in a 1.5 ml Eppendorf tube for each electroporation reaction. Cells were centrifuged at 300xg for 6 minutes at 4°C and supernatant was carefully removed. Cells were resuspended in the nucleofection reagent (82 µl nucleofection V solution and 18 µl supplement solution for each reaction) and finally mixed with 3.5 µg of plasmid (volume of the cell pellet and plasmid should not be more than 50% of the volume of transfection reagent mix). The mixture of cells with plasmid was then transferred to the electroporation cuvette (provided with the nucleofection kit). Electroporation was performed with the Nucleofector™ 2b Device (Lonza) using the program T-029. Once the electroporation reaction was complete, 500 µl of fresh complete OSC culture media was added to the cuvette. Cells were transferred from the cuvette to a single well of a 6-well plate (already containing 1.0 ml complete medium in each well) using sterile plastic Pasteur pipette. Note that electroporation causes lot of cell death and they usually form a white precipitate in the cuvette. While transferring the transfected cells to culture plate, this white layer of dead cells must be avoided. Transfection efficiency was monitored with a plasmid expressing EGFP (pAc5.1B-EGFP, Addgene Plasmid #21181) and number of EGFP-positive cells was counted under a fluorescence microscope. We routinely obtain transfection efficiency of 70-80%. Cells were harvested after 48 hours post transfection.

Female fly extract preparation protocol

Young female flies (maximum 3-4 days old) were collected. They were homogenized in cold Shields and Sang M3 Insect Medium (6.8 ml medium/1 g of female flies) on ice. The total lysate was then centrifuged at 16000xg for 15 minutes at 4℃. Supernatant was collected in a new tube and heat inactivated in a pre-warmed water bath at 60℃ for 5 minute (gently mix the lysate a few times during heat inactivation). The heat-inactivated supernatant was transferred on ice for at least 5 minutes and then centrifuged again at 16000xg for 30 minutes at 4℃. Supernatant was collected, filter sterilized (0.22 µ filter) and stored in aliquots of 1.5ml at -80℃. For preparation of 100 ml complete OSC medium, 2.5 ml of the fly extract was added to the medium.

**Purification of recombinant Armi or Armi-Piwi complex**

The MultiBac system [[10](#_ENREF_10)] was used to clone genes into either acceptor or donor vectors, these vectors were then fused together to generate one expression plasmid by Cre-Lox recombination strategy. Armi coding sequence was cloned into either pACEBac2-Sumo acceptor vector or pIDS-Sumo donor vector. The MBP-tagged Piwi was cloned into pACEBac2 acceptor vector.

The following constructs were prepared.

Armitage (Armi; 1-1188 aa), in NheI, SphI sites of pACEBac2-Sumo or pIDS-Sumo vectors.

Armi^DQAG^ mutant with E863Q mutation, in NheI, SphI sites of pACEBac2-Sumo vector.

Piwi (1-843 aa) in NheI, SphI sites of pACEBac2-MBP vector.

Insect cell expression and purification

For production of recombinant proteins in the insect cells the following ovary-derived cells were used: Sf21 or Sf9 from Fall Army worm *Spodoptera frugiperda* or High Five (Hi5) from the cabbage looper, *Trichoplusia ni*. Expression of desired coding sequences was carried out with the use of recombinant Baculoviruses. Either single or multiple coding sequences were integrated into the Baculovirus genome using the MultiBac protein expression system [[10](#_ENREF_10)]. The coding sequence for *Drosophila* Armitage (Armi) was isolated by RT-PCR from fly ovarian total RNA, while the codon-optimized DNA sequence was commercially synthesized (Shanghai ShineGene Molecular Biotech,Inc.).

For Armi^DQAG^ mutant, the catalytic residue glutamic acid 863 was mutated to glutamine from DEAG to DQAG. To mutate glutamic acid to glutamine, the DNA codon GAA for glutamic acid has been changed to CAA for glutamine. The forward and reverse primers with mutation carrying the nearby 12 nucleotides on either side are designed for PCR-based mutagenesis.

For: GGCCATTTCACTCACGTGCTGTTCGATCAAGCTGGTCAATGC

Rev: GCATTGACCAGCTTGATCGAACAGCACGTGAGTGAAATGGCC

The following proteins were expressed in insect cells:

pACEBac2-Sumo (6xHis-Strep-SUMO-TEV) -DmArmitage FL (1-1188aa)

pACEBac2-Sumo (6xHis-Strep-SUMO-TEV) -DmArmitage (1-1188 aa) E863Q mutant

pACEBac2-MBP-Piwi + pIDS-His-Strep-SUMO-DmArmitage

The Hi5 insect cells expressing Armi were lysed in buffer [50 mM Tris-HCl pH 8.0, 500 mM NaCl, 5 mM 2-Mercaptoethanol, 20 mM Imidazole, 0.5% Tween-20, 10% Glycerol and protease inhibitor (Roch)], and the clarified lysate was mixed with Ni-NTA beads. The elution is then transferred to StrepTrap HP column (GE Healthcare, cat. No. 28-9075-46). The tagged protein was further purified over the ion exchange column (HiTrap^TM^ Q HP, GE healthcare, 17-1154-01). The relatively pure fractions containing the recombinant proteins were further purified by gel filtration chromatography (Superdex S200 10/300GL, GE healthcare, cat. No. 17-5175-01) (S2A Fig). The pure fractions of 6xHis-Strep-SUMO-Armi were used for helicase assay without removal of the tag. The mutant version of the protein was also prepared similarly. All proteins eluted in similar fractions during gel filtration chromatography, indicating that the mutations did not grossly affect protein structure. Purified proteins were confirmed by mass spectrometry (EMBL proteomics core facility, Heidelberg).

For co-expression of Armi and Piwi, insect cells expressing them were lysed in buffer [50 mM Tris-HCl pH 8.0, 150 mM NaCl, 5 mM 2-Mercaptoethanol, 10% Glycerol and protease inhibitor(Roch)], and the clarified lysate was subjected to tandem affinity-purification via StrepTrap column and then MBPTrap column (GE healthcare, 29-0486-41) purifications. The eluted complex was resolved by SDS-PAGE and examined by Coomassie staining to reveal two prominent bands (Fig 3D). These were identified by mass spectrometry (EMBL proteomics core facility, Heidelberg), to be tagged versions of Armi and Piwi, which we also confirmed by Western blotting with anti-His and anti-MBP antibodies. We note that preparations of MBP-Piwi alone are always very dirty with multiple bands. It also elutes in the void during gel filtration chromatography, indicating aggregation of the protein, perhaps due to its unloaded status. When co-expressed with His-Strep-SUMO-Armi and purified sequentially over StrepTrap and MBPTrap, we obtain preparations containing very pure MBP-Piwi and tagged-Armi.

**RNA unwinding assay**

RNA unwinding reaction was performed as described [[11](#_ENREF_11), [12](#_ENREF_12)], with some modifications. Single stranded RNA oligos were chemically synthesized (Microsynth, CH) and sequences are given below. The region of complementarity is underlined. Substrates for RNA unwinding assay were prepared by annealing 5ʹ radio-labelled top strand with cold bottom strand.

Top strand 1: 5ʹ-AGCACCGUAAAGACGC-3ʹ, **RP RNA18**

Bottom strand 1: 5ʹ-GCGUCUUUACGGUGCUUAAAACAAAACAAAACAAAACAAA-3ʹ **RP RNA19**

Bottom strand 2: 5ʹ-AAACAAAACAAAACAAAACAAAAUAGCACCGUAAAGACGC-3ʹ **RP RNA20**

Top strand 2: 5ʹ-GCGUCUUUACGGUGCU-3ʹ **RP RNA21**

Briefly, 0.6 µl of top strand (100µM) was radiolabelled with 6.5 µl of (γ-^32^P)-ATP in a 10 µl reaction using T4 PNK (15 units, Thermo Fisher) at 37°C for 45 minutes. Radiolabelled RNA was separated on 15% urea polyacrylamide gel and correct size RNA band was cut out from the gel and eluted in 400µl of 0.4M NaCl at 25°C overnight on a thermomixer. Next day, eluted RNA was precipitated with 2.5 volumes of 100% ethanol and finally re-suspended in 2 µl of 10X duplex annealing buffer (100 mM MOPS, pH 6.5, 10 mM EDTA, 0.5 M KCl). The cold bottom strand RNA (1.0 µl from 100µM stock) was added to the top strand radiolabelled RNA and the final volume was made up to 20 µl. RNA mix was heated at 95°C for 5 minutes and then left to cool down to 37°C on its own. The RNA duplex was resolved on non-denaturing polyacrylamide gel in the cold room and the RNA band corresponding to duplex RNA was cut out and eluted from the gel, precipitated and re-suspended in 40µl of RNase-free water. RNA unwinding reaction was performed with recombinant Armitage (wild type or ATP hydrolysis mutant Armi^DQAG^) and 2.0 µl of RNA duplex in helicase reaction buffer [40 mM Tris-HCl, pH 7.5, 50 mM NaCl, 0.5 mM MgCl_2_, 2 mM DTT and 0.01% (v/v) NP-40]. Reaction mix with protein and RNA substrate was incubated at 37°C for 10 minutes without ATP. The unwinding reaction was started by adding 1mM ATP (final concentration in 30 µl reaction mix) and samples were collected at different time point. Reactions were stopped by adding stop buffer (1 % SDS, 50 mM EDTA, 0.1 % xylene cyanol, 0.1 % bromophenol blue, and 20 % glycerol). The samples were finally analyzed on non-denaturing TBE-polyacrylamide gel in cold room. The gel was dried and exposed to Storage Phosphor screen overnight. The screen was scanned with typhoon scanner (GE Health).

**Immunofluorescence**

Ovaries from 3-4 days old *Drosophila* from each genotype were dissected in 1X PBS. For staining of germline-expressed proteins, ovaries were fixed in salt solution (0.4% NaCl, 0.3% Triton X-100) in an eppendorf tube at 92°C for 5 minutes followed by addition of equal volume of ice-cold salt solution. For staining the follicle-cells-expressed protein, ovaries were fixed in 4% paraformaldehyde in PBS for 30 minutes on a shaker. Fixed ovaries were washed three times, 10 minutes each, with 1.0 ml PBT (1X PBS, 0.1% Triton X-100) on shaker. The ovaries were extracted in PBT (1X PBS, 1% Triton X-100) for 1 hour on shaker. After extraction, ovaries were blocked in blocking buffer (1X PBS, 0.3% Triton X-100, 0.5% BSA) for 1-2 hours and then they were incubated with primary antibodies in the blocking buffer overnight on a shaker in cold room. Next day, they were washed twice with the blocking buffer, 20 minutes each and then blocked with a new blocking buffer (1X PBS, 0.1% Triton X-100, 10% goat serum) for one hour. Secondary antibodies conjugated with Alexa Fluor dye (Life tech, Alexa Fluor 488 and 594) were added at a dilution of 1:300 and incubated for 2-3 hours in a dark chamber. The secondary antibodies were removed after 2-3 hours and ovaries were incubated with DAPI 1:2500 dilution (stock is 1mg/ml) in PBS for 5 minutes. Finally, ovaries were washed twice, 20 minutes each, with PBT (1X PBS, 0.1% Triton X-100) and then mounted in mounting media. Images were captured on Zeiss LSM 780 confocal microscope.

**Immunoprecipitations**

Approximately 48 hours post-transfection of OSC, culture medium was removed and cells were washed twice with 2.0 ml cold 1XPBS. 1.0 ml of cold lysis buffer [50 mM Tris-base pH 8.0, 150 mM NaCl, 5 mM MgCl_2_, 1 mM DTT, 10% glycerol, 1% TritonX-100, 0.5% Sodium deoxycholate, 10μl/ml vanadyl ribonucleoside complex (Sigma, Cat. no. 94742) and Protease Inhibitor cocktail (Roche, Cat. no. 05056489001)] was directly added to each well. Cells were lysed by pipetting several times. Lysed cells were collected and centrifuged at 16000xg for 15 minutes at 4℃ to remove insoluble cell debris. Endogenous Piwi was immunoprecipitated using specific antibodies immobilized on Protein G Sepharose beads. Cleared cell lysate was incubated with antibody-bound Protein G Sepharose beads on a rotating wheel (5 rpm rotation speed) in the cold room. After 4 hours, beads were washed five times with wash buffer (10 mM Tris pH 8.0, 150 mM NaCl, 0.01% NP-40, 5% glycerol). Beads were transferred to a new tube during last wash step. Immunoprecipitated small RNAs were eluted by proteinase K digestion (300 ul of 1X proteinase K buffer and 20 μg of proteinase K) at 42℃ for 10 minutes followed by phenol-choloroform extraction and ethanol precipitation.

For small RNA immunoprecipitation from *Drosophila* ovary, the tissue (abdomen of ~ 50 female flies/immunoprecipitation) was homogenized in lysis buffer (see above for details) using a glass homogenizer. Lysate was cleared by centrifugation at 16000xg for 15 minutes at 4℃. The cleared lysate was used for immunoprecipitation using different antibodies for Piwi, Aub and Ago3.

**Small RNA libraries**

All small RNA libraries were prepared with piRNAs associated with endogenous PIWI proteins, which were isolated by immunoprecipitations. For *Drosophila* ovarian PIWI immunoprecipitations, RNAs were isolated from complexes and then resolved by 15% urea-PAGE. RNAs migrating at ~24-28 nt size-range were carefully gel-eluted by avoiding the contaminating 30 nt-sized 2S rRNA frequently found in these samples. For OSC Piwi immunoprecipitations, RNAs isolated were directly used for library preparation. Small RNA libraries (barcoded at 3′ end) were prepared using NEBNext® Multiplex Small RNA Library Prep Set for Illumina® (NEB Catalogue No. E7300). The libraries were sequenced with the Illumina HiSeq 2000 platform (EMBL Gene Core facility) for 50 cycles.

**Bioinformatic analysis of small RNA libraries**

Reads were sorted into individual libraries based on the barcodes and the 3′ adapter sequences were clipped using cutadapt (DOI:http://dx.doi.org/10.14806/ej.17.1.200). Only reads which are at least 15 nucleotides in length were left for subsequent analysis and the independent replicated libraries were merged together. Reads were then aligned to the desired reporter sequence using bowtie [[13](#_ENREF_13)] allowing no mismatches. Analysis was as previously described [[1](#_ENREF_1)]. Briefly, the piRNA coverages (for each nucleotide the number of encompassing piRNAs) were calculated, normalized to 1 million of library reads and plotted along the reporter as reads per million (rpm). When two coverages were compared, the log2 ratio of normalized coverages was plotted (the raw 0 read coverage values were substituted by 1 to enable the calculation of the ratios). To analyze whether the piRNAs are produced in a phased manner, we calculated the fractions of piRNA pairs located in a specific distance. The score was calculated either for 5' to 5' or 3' to 5' distances. The score for specific distance Δ was calculated as: score(Δ) = Σmin(M(i),N(i+Δ)) where M(i) is the count of produced piRNAs (in rpm) with the specified end at a particular position i and N(i+Δ) is the count of piRNAs on the same strand which have their specified end position at i + Δ.

**Supplemental References**

1. Homolka D, Pandey RR, Goriaux C, Brasset E, Vaury C, Sachidanandam R, et al. PIWI Slicing and RNA Elements in Precursors Instruct Directional Primary piRNA Biogenesis. Cell Rep. 2015;12(3):418-28.

2. Saito K, Ishizu H, Komai M, Kotani H, Kawamura Y, Nishida KM, et al. Roles for the Yb body components Armitage and Yb in primary piRNA biogenesis in Drosophila. Genes Dev. 2010;24(22):2493-8.

3. Handler D, Olivieri D, Novatchkova M, Gruber FS, Meixner K, Mechtler K, et al. A systematic analysis of Drosophila TUDOR domain-containing proteins identifies Vreteno and the Tdrd12 family as essential primary piRNA pathway factors. EMBO J. 2011;30(19):3977-93.

4. Saito K, Inagaki S, Mituyama T, Kawamura Y, Ono Y, Sakota E, et al. A regulatory circuit for piwi by the large Maf gene traffic jam in Drosophila. Nature. 2009;461(7268):1296-9.

5. Pillai RS, Artus CG, Filipowicz W. Tethering of human Ago proteins to mRNA mimics the miRNA-mediated repression of protein synthesis. RNA. 2004;10(10):1518-25.

6. Murota Y, Ishizu H, Nakagawa S, Iwasaki YW, Shibata S, Kamatani MK, et al. Yb integrates piRNA intermediates and processing factors into perinuclear bodies to enhance piRISC assembly. Cell Rep. 2014;8(1):103-13.

7. Bateman JR, Lee AM, Wu CT. Site-specific transformation of Drosophila via phiC31 integrase-mediated cassette exchange. Genetics. 2006;173(2):769-77.

8. Bischof J, Maeda RK, Hediger M, Karch F, Basler K. An optimized transgenesis system for Drosophila using germ-line-specific phiC31 integrases. Proc Natl Acad Sci U S A. 2007;104(9):3312-7.

9. Venken KJ, He Y, Hoskins RA, Bellen HJ. P[acman]: a BAC transgenic platform for targeted insertion of large DNA fragments in D. melanogaster. Science. 2006;314(5806):1747-51.

10. Bieniossek C, Imasaki T, Takagi Y, Berger I. MultiBac: expanding the research toolbox for multiprotein complexes. Trends Biochem Sci. 2012;37(2):49-57.

11. Abdelhaleem M. Helicases: an overview. Methods Mol Biol. 2010;587:1-12.

12. Gregersen LH, Schueler M, Munschauer M, Mastrobuoni G, Chen W, Kempa S, et al. MOV10 Is a 5' to 3' RNA helicase contributing to UPF1 mRNA target degradation by translocation along 3' UTRs. Mol Cell. 2014;54(4):573-85.

13. Langmead B, Trapnell C, Pop M, Salzberg SL. Ultrafast and memory-efficient alignment of short DNA sequences to the human genome. Genome Biol. 2009;10(3):R25.
